# Synergistic Antibacterial Activity of Designed Trp-Containing Antibacterial Peptides in Combination With Antibiotics Against Multidrug-Resistant *Staphylococcus epidermidis*

**DOI:** 10.3389/fmicb.2019.02719

**Published:** 2019-11-25

**Authors:** Dejing Shang, Yue Liu, Fengquan Jiang, Fangyu Ji, He Wang, Xue Han

**Affiliations:** ^1^School of Life Sciences, Liaoning Normal University, Dalian, China; ^2^Liaoning Provincial Key Laboratory of Biotechnology and Drug Discovery, Liaoning Normal University, Dalian, China; ^3^Clinical Laboratory Department of the First Affiliated Hospital, Dalian Medical University, Dalian, China

**Keywords:** synergism, Trp-containing peptide, antibiotics, membrane, biofilm

## Abstract

Multidrug resistance among various bacterial strains is leading to worldwide resistance to a wide range of antibiotics. Combination therapy involving current antibiotics and other biological or chemical molecules represents an attractive novel strategy. In this study, we investigated the synergistic antibacterial activity of a series of Trp-containing antimicrobial peptides (AMPs) with four classes of traditional chemical antibiotics that are inactive against multidrug-resistant *Staphylococcus epidermidis* (MRSE) *in vitro* and *in vivo*. Among the antibiotics that we studied, penicillin, ampicillin and erythromycin showed a distinct synergistic effect in combination with all of the Trp-containing AMPs, represented by a fractional inhibitory concentration index (FICI) of <0.5. The antibacterial activities were noticeably improved, with 32-to 64-fold reductions in the MIC values for ampicillin and 16- to 32-fold reductions in the MIC values for erythromycin and penicillin. Tetracycline showed synergistic activity with only I1WL5W but additive activity with L11W, L12W, and I4WL5W. Ceftazidime exhibited additive activity with the Trp-containing peptides. In addition, the antibiotics in combination with the peptide significantly inhibited biofilm formation by MRSE 1208. A mechanistic study demonstrated that the Trp-containing peptides, especially I1WL5W and I4WL5W, which contain two tryptophan residues, disrupted bacterial inner and outer membranes, which promoted antibiotic delivery into the cytoplasm and access to cytoplasmic targets; however, L11W and L12W may have increased intracellular antibiotic concentrations by decreasing *bla*Z, *tet*(m) and *msr*A expression. Importantly, strong synergistic activity against the MRSE 1208 strain was observed for the combination of I1WL5W and penicillin in a mouse infection model. Thus, the combination of AMPs and traditional antibiotics could be a promising option for the prevention of acute and chronic infections caused by MRSE.

## Introduction

Over the last few decades, a growing number of multidrug-resistant bacterial species have dramatically increased because of the widespread use of antibiotics, which has become a major problem for health care centers worldwide. Although the development of novel antibiotics with different mechanisms of action has been carried out, processes of novel drug discovery and development usually span 10–17 years, and the success rate is lower than 10% (Ashburn and Thor, 2004; Spellberg et al., 2013; Almaaytah et al., 2016). Furthermore, new resistant strains will inevitably emergence again as the widespread use and misuse of novel drugs with a single target of action. Therefore, reviving the therapeutic potential of existing antibiotics via effective combinations with other biological or chemical molecules represents an approach to improve antibacterial activity and prevent the emergence of resistance. Combination therapy with antibacterial agents can reduce the dose of drugs to minimize adverse effects and thus be a way to overcome problems with toxicity and the development of resistance (Soren et al., 2015).

Antimicrobial peptides represent promising alternative agents to conventional antibiotics because of their high effective antimicrobial activity against a broad spectrum of microorganisms (Zhou et al., 2010; Fjell et al., 2011; Pasupuleti et al., 2011; Abd-El-Aziz et al., 2015). It is generally known that most AMPs target the cell membrane, and the ability to disrupt the membrane endows the antimicrobial activity of AMPs. However, low antibacterial target selectivity and high toxicity to mammalian cells has hindered the development of AMPs as successful therapeutics for clinical usage (Marr et al., 2006). Previous studies have demonstrated that the combination of some synthetic short and cationic peptides with conventional antibiotics exceeds the antibacterial activities of the individual drugs and helps prevent the development of resistance in microorganisms (Maisetta et al., 2009; Kajal et al., 2015; Regmi et al., 2017a). Synergy between AMPs and antibiotics also reduces peptide toxicity during treatment of *Mycobacterium* infections, similar to other peptide-antibiotic combinations reported previously (Khara et al., 2014). The generic membrane-disrupting activity of AMPs helps give conventional small molecule antibiotics increased access to the cell. A synergistic effect between peptides and traditional antibiotics can be a potential option to improve the effectiveness of antimicrobial agents, increase bacterial killing and prevent the emergence of the development of both antibiotic and peptide resistance.

Antimicrobial peptides containing tryptophan (Trp) residues display more potent antimicrobial activity because of the interaction of the aromatic side chain of Trp residues with the interfacial region of a membrane by forming hydrogen bonds with a dipole moment of ∼21 D (Khandelia and Kaznessis, 2007). These special features make Trp-containing peptides partition into the bilayer interface. In our previous study, a series of Trp-containing peptides were designed and synthesized by substituting Ile or Leu residues at sites 1, 4, 5, 11, and 12 with one Trp residue (named I1W, I4W, L5W, L11W, and L12W, respectively) or by substituting two Trp residues (I1WL5W and I4WL5W) based on the structure of a peptide derived from the frog skin peptide temporin-1CEb (Shang et al., 2009; Bi et al., 2013, 2014). These peptides have been found to induce low levels of hemolysis (HC_50_ > 500 μM), and low cytotoxicity on mouse macrophage RAW264.7, human renal epithelial cell 293T, human macrophage THP-1 and human colon adenocarcinoma Caco-2 cells for L11W and l12W (data not shown). L11W and L12W exhibited moderate antibacterial activity against gram-positive bacteria but no or low activity against gram-negative bacteria. I1WL5W and I4WL5W showed high activity against a broad spectrum of microorganisms. The Trp residues show a strong membrane-disruptive activity, and this property endows Trp-containing peptides with the unique ability to interact with the surface of bacterial cell membranes. In this study, we aimed to elucidate whether the combination of Trp-containing AMPs with commercially available antibiotics provides a synergistic action and thus creates a more effective approach to the treatment of multidrug-resistant bacteria. The multidrug-resistant *S. epidermidis* 1208 (MRSE 1208) strain was isolated from the clinical specimens of a patient and shows resistance to ampicillin, amoxicillin, cefazolin, ciprofloxacin, erythromycin, imipenem, levofloxacin, penicillin, ceftazidime and rifampicin; moderate sensitivity to tetracycline and vancomycin; and sensitivity to chloramphenicol and sulfamethoxazole. Five commercial antibiotics, penicillin, ampicillin, erythromycin, ceftazidime, and tetracycline, were chosen to investigate the synergistic effect of combination therapies against the MRSE 1208 strain by using the checkerboard method and the required therapeutic doses of five conventional antibiotics. In addition, the potential action mode of synergy *in vitro* and *in vivo* was investigated.

## Materials and Methods

### Peptide Synthesis

The Trp-containing peptides (purity ≥95%) used in this study, L11W, L12W, I1WL5W, and I4WL5W were purchased from GL Biochemistry Inc. (Shanghai, China). Amino acid sequences and physical characteristics for peptides are shown in Table 1.

**TABLE 1:** Biological characteristics of the Trp-containing antimicrobial peptides (Bi et al., 2013, 2014).

**Peptide**	**Amino acid sequence**	**Amphipathicity**	**Hydrophobicity**	**Molecular mass (Da)**
L-K6	IKKILSKIKKLLK-NH_2_	0.83	12.45	1663.0
L11W	IKKILSKIKKWLK-NH_2_	0.82	13.33	1626.1
L12W	IKKILSKIKKLWK-NH_2_	0.83	13.33	1626.1
I1WL5W	WKKIWSKIKKLLK-NH_2_	0.83	13.15	1698.2
I4WL5W	IKKWWSKIKKLLK-NH_2_	0.82	13.15	1698.2

*The hydrophobicity of the peptide calculated using the hydrophobicity scale (Mant et al., 2009) was the total hydrophobicity (sum of the hydrophobicity indices for all residues) divided by the number of residues. Amphipathicity was determined by calculation of the hydrophobic moment (Eisenberg et al., 1982; Carver and Bleasby, 2003).*

### Bacterial Strains

The multidrug-resistant *S. epidermidis* strain 1208 (MRSE 1208) was obtained from a clinical specimen admitted to the First Affiliated Hospital of Dalian Medical University. The MRSE 1208 strain exhibited multidrug resistance to ampicillin, amoxicillin, cefazolin, ciprofloxacin, erythromycin, imipenem, levofloxacin, penicillin, ceftazidime and rifampicin. Identification and antibiotic susceptibility testing of the strains were performed by the Clinical Laboratory Department of the First Affiliated Hospital of Dalian Medical University. The bacterial strain *S. epidermidis* (CICC 23664) was acquired from the China Center for Industrial Culture Collection.

### Determination of Minimal Inhibitory Concentrations

Minimum inhibitory concentrations (MICs) of peptides and antibiotics were determined using a standard microdilution method in 96-well microtiter plates as described previously (Bi et al., 2013). Briefly, the peptides and antibiotics were two-fold serially diluted to concentrations between 1.56 and 200 μM for peptide, and 0.78 and 200 μM for erythromycin, tetracycline, penicillin, ampicillin, and ceftazidime. 100 μL of log-phase bacterial cultures (at a final concentration of 1 × 10^5^ colony-forming units (CFU)/ml) were filled into each well of a microtiter plate. Subsequently, 100 μL of the peptide or antibiotic solution was added to the bacterial cultures and incubated at 37°C overnight. The optical density (OD) at 600 nm (OD600) for each sample was recorded using a microtiter plate reader (Varioskan Flash Microplate Reader, Thermo Scientific Co., Beijing). The MIC was defined as the lowest peptide concentration that inhibited 95% of the bacterial growth.

### Checkerboard Assay

Combinations of the peptide with the antibiotics were evaluated by the checkerboard assay according to Berditsch et al. (2015). The peptides and antibiotics were serially diluted two-fold in a horizontal orientation and a vertical orientation, respectively, in a 96-well microtiter plate. The concentrations started from their respective MICs and were then subjected to two-fold serial dilutions. Then, 100 μl of single peptides or a peptides/antibiotics combination were inoculated with 100 μl of the bacterial suspension (1 × 10^5^ CFU/ml). MH medium was used as a negative control, and the peptide or antibiotic alone was used as a positive control. After incubation at 37°C overnight, the OD600 value was measured with a microplate plate reader (Varioskan Flash Microplate Reader, Thermo Scientific Co., Beijing). Synergistic interactions were expressed as the fractional inhibitory concentration index (FICI), which is calculated as follows: FICI = FIC_a_ + FIC_b_, where FIC_a_ and FIC_b_ are the MICs of the peptides in the combination divided by the MICs of the peptides alone and the MICs of the antibiotics in the combination divided by the MICs of the antibiotics alone, respectively. FICI ≤ 0.5, 0.5 < FICI ≤ 1.0 and 1.0 < FICI ≤ 2.0 were defined as a synergy, addition and indifference, respectively (Hollander et al., 1998; Yoon et al., 2004). The results were collected from at least six independent experiments, and median FICI values were used in the analysis.

### Hemolysis and Cytotoxicity Assay

The hemolysis of the peptides in combination with penicillin were determined according to previous method (Shang et al., 2012). Briefly, 2 × 10^7^ erythrocytes collected from healthy human blood were washed three times with 0.9% NaCl and then incubated with different concentration of penicillin in combination with 1/4 MIC peptides at 37°C water bath for 180 min. The sample suspension was obtained by centrifugation at 300 × *g* for 5 min. The OD_540_ of sample suspension was measured by spectrophotometer. Erythrocyte incubated in 0.1% Triton X-100 (100% hemolysis) or in 0.9 NaCl (0% hemolysis) were used as the positive and negative controls, respectively. The percentage of hemolysis was calculated as follows:% hemolysis = 100 × [(Abs in the sample – Abs in 0.9% NaCl)/(Abs in 0.1% Triton X-100 – Abs in 0.9% NaCl)].

The cytotoxicity of the peptides in combination with penicillin on the viability of the human renal epithelial cell 293T was evaluated by using MTT assay (Wang et al., 2012). Cells were treated with different concentration of penicillin in combination with 1/4 MIC peptides in 96-well plate for 24 h. Then, 10 μl of 5 mg/ml MTT solution reagent was added to each well and the plates were incubated for 4 h. The supernatant was removed and 100 μL of DMSO was added to each well to dissolve the formazan crystals. The OD_490_ was read with microplate reader. Results are expressed as a percentage of cell viability (cell viability rate) calculated as the ratio of the mean of OD obtained for each condition to that of control condition (negative control). All experiments were repeated three times.

### Biofilm Assay

Adhesion and formation of biofilm of the MRSE 1208 strain were examined according to our previous work (Shang et al., 2014). Briefly, log-phase cultures were diluted to 2 × 10^5^ CFU/ml after washing twice. A total of 100 μl of the bacterial solution was placed into a 96-well microtiter plate containing 100 μl of medium (non-treated control), different concentration of antibiotics as a single drug control, or 1/4 × MIC of peptides and different concentrations of antibiotics in combination. After incubation at 37°C for 1 or 24 h, planktonic cells were carefully removed by centrifugation, and then the wells with biofilms were washed three times and fixed. Biofilms were stained with 0.1% (w/v) crystal violet dye (CV) for 5 min and quantified by reading at 590 nm in a microtiter plate reader (Varioskan Flash Microplate Reader, Thermo Scientific Co., Beijing). The biofilm biomass was calculated using the following formula: OD590 of the sample/OD590 of the non-treated control. The biofilm biomass at 1 and 24 h represents the adhesion and formation of biofilm, respectively.

One-day-old biofilms were prepared according to our previously described procedure (Shang et al., 2014). The biofilms were grown for 24 h and then washed with phosphate-buffered saline (PBS) and incubated with peptides or antibiotics as a single drug control, 1/4 × MIC of peptides and different concentrations of antibiotic in combination at 37°C for 24 h. LB liquid medium was used as a negative control. The biofilms were then fixed, stained and quantified as described above. All experiments were repeated three times.

### Real-Time Quantitative PCR Analysis

Midlog-phase MRSE 1208 cells (1 × 10^8^ CFU/ml) were incubated with peptide at concentrations of 1.25, 2.5, 5.0, 10.0, and 20.0 μM at 37°C for 18 h. The bacterial cells were collected, washed and digested in a 10 mg/ml lysozyme solution. Total RNA was extracted with TRIzol reagent (Invitrogen, Shanghai, China) and reverse transcribed into cDNA using a Super Script^TM^ III kit (Invitrogen, Shanghai, China). Real-time PCR amplification was performed using the ABI Prism Q5 sequence detection system (Applied Biosystems, United States). PCR primers for *bla*Z, *tet(m), mec*A and *msr*A are presented in Supplementary Table S1. The reaction conditions were as follows: 94°C for 30 s, followed by 40 cycles of 94°C for 5 s, 55°C for 15 s, and 72°C for 10 s. Gene expression levels were normalized using 16S rRNA as an internal standard, and fold changes were calculated by the comparative cycle threshold (Ct) method.

### Inner Membrane Depolarization Assay

Bacterial membrane depolarization was measured using a membrane potential-sensitive fluorescent dye, DiSC3-5 (Sigma–Aldrich, Shanghai, China), that changes its fluorescence intensity in response to changes in transmembrane potential. Midlog-phase bacterial cells were centrifuged at 3000 × *g* for 10 min, washed twice with 5 mM HEPES (pH 7.2) containing 20 mM glucose and 100 mM KCl, and resuspended in the same solution at a final concentration of 2 × 10^6^ CFU/ml. DiSC3-5 was added to a final concentration of 4 μM in a black NBS microplate, and the change in dye fluorescence was continuously monitored at an excitation wavelength of 622 nm and an emission wavelength of 670 nm at 30 s intervals using a Varioskan Flash Microplate Reader (Thermo Scientific Co., Beijing, China). When a stable reduction in the fluorescence occurred because of quenching of the accumulated dye in the membrane interior, DiSC3-5 had achieved maximal uptake by the bacteria. The peptides at a final concentration of 1/4, 1, or 4 times their MIC; 3.125 μM penicillin (1/32 × MIC); and the combination of peptides (1/4 × MIC) and 3.125 μM penicillin were added, and the membrane potential was determined by the change in fluorescence intensity. Triton X-100 was used as a control reference. The measurements were repeated at least three times for each dye concentration.

### Outer Membrane Permeability Assay

Outer membrane permeability was analyzed using 1-N-phenylnaphthylamine (NPN, Solarbio, Beijing, China) dye. Midlog-phase bacteria were centrifuged, washed twice and resuspended in 10 mM sodium phosphate buffer to 2 × 10^6^ CFU/ml. NPN was added at a final concentration of 10 μM to 500 μl of the bacterial cells, and the basal fluorescence intensity was recorded as a control (excitation wavelength of 350 nm/emission maximum of 420 nm). The peptides at a final concentration of 1/4, 1, or 4 times their MIC; 3.125 μM penicillin; and the combination of peptides (1/4 × MIC) and 3.125 μM penicillin were added to the cells with NPN. After 60 min, fluorescence intensity was recorded. The measurements were repeated at least three times for each dye concentration.

### Scanning Electron Microscopy

Scanning electron microscopy (SEM) was used to examine the effect of the peptide/penicillin combination on the cellular morphology of the MRSE 1208 strain. A log-phase bacterial culture (at final concentration 1 × 10^8^ CFU/ml) was treated with 1/4 × MIC of L12W, 1/4 × MIC of I1WL5W, 3.125 μM penicillin, or the combination of I1WL5W/penicillin or L12W/penicillin at 37°C for 1 h. The bacterial cultures were washed three times and fixed with warm fixative solution (2.5% buffered glutaraldehyde in 0.1 M cacodylate buffer, pH 7.2) at 4°C for 2 h, followed by postfixation in 1% buffered osmium tetroxide for 2 h and dehydration in an ascending series of ethanol concentrations. The samples were lyophilized, coated with approximately 5 nm of gold/palladium, and then observed under a scanning electron microscope (Hitachi SU8010, Hitachi, Japan).

### Establishment of an MRSE 1208 Infection Model

A mouse wound infection model was established to assess the *in vivo* antimicrobial activity of the penicillin/I1WL5W combination against MRSE 1208 (Bracho et al., 2009; Otto, 2014). A total of 96 female Kunming mice (20 ± 1 g) were purchased from the Laboratory Animal Center of Dalian Medical University and were randomly divided into four groups, with 24 mice in each group. The procedures used to produce wound infections have been described previously (Sun et al., 2015; Dong et al., 2018). The protocol was approved by the Committee of Care and Use of Laboratory Animals in Liaoning Normal University. Briefly, mice were anesthetized with an intraperitoneal injection of sodium pentobarbital (30 mg/kg), and then the dorsal hair was shaved. A full-thickness skin section was excised, and then the wound was infected with an MRSE 1208 culture (2 × 10^8^ CFU/ml). On the third day of infection, the wounds were treated with physiological saline (the negative control), I1WL5W (1/4 × MIC) alone, the combination of I1WL5W (1/4 × MIC)/6.25 μM penicillin and the combination of I1WL5W (1/4 × MIC)/12.5 μM penicillin every 12 h for 6 days. At 0, 3 or 6 days of the treatment of the samples, eight mice from each group were sacrificed, and blood was collected from mice and rapidly centrifugated at 5,000 × *g* for 10 min at 4°C. The supernatants were stored at -20°C for enzyme-linked immunosorbent (ELISAs) analysis. The wounded skin was aseptically excised, weighed and homogenized in distilled water to count bacterial CFU in the skin. Another skin sample was observed by optical microscopy (DM750, Leica, Germany) after being fixed in 10% formalin, buffered in phosphate-buffered saline, and processed for hematoxylin and eosin (H&E) staining. Interleukin-6 (IL-6) and tumor necrosis factor (TNF-α) in mouse serum were determined by ELISAs (Rapidbio, Shanghai, China) according to the manufacturers’ protocol.

### Statistical Analysis

All the experiments were performed in triplicate. The results are generally expressed as the means and standard errors. A paired Student’s *t*-test was used to test for significance. Significance is indicated as fellows: ^∗^for *p* < 0.05, ^∗∗^for *p* < 0.01.

## Results

### Antibacterial Activity of Trp-Containing Peptides

The antibacterial activity of the Trp-containing peptides against the MRSE 1208 strain is listed in Table 2. All of the peptides exhibited strong antibacterial activity against the MRSE 1208 strain, with MIC values of 3.125–12.5 μM. These four peptides have the same net charge of +7, the same length of 13 residues and a highly similar sequence. However, I1WL5W and I4WL5W showed higher effectiveness than L11W and L12W against both the multidrug-resistant MRSE 1208 strain and the susceptible *S. epidermidis* CICC 23664 strain. These Trp-containing peptides, except L12W, exhibited weaker antibacterial activity against the multidrug-resistant *S. epidermidis* strain (MRSE 1208) than against the susceptible *S. epidermidis* CICC 23664 strain, suggesting that MRSE 1208 was slightly resistant to these peptides. L12W, a peptide with one tryptophan residue at position 1 in the carboxyl terminus of the peptide, had the same antibacterial activity against the MRSE 1208 strain and *S. epidermidis* CICC 23664 strain. Interestingly, although L12W had weak activities against both susceptible gram-positive and gram-negative bacteria (Bi et al., 2013), we found that the clinically isolated multidrug-resistant *Klebsiella pneumoniae, Enterobacter cloacae*, *Staphylococcus aureus, Enterococcus faecalis, Acinetobacter baumannii, Enterobacter aerogenes*, and *Pseudomonas fluorescens* showed no drug resistance to L12W, among of the nine designed Trp-containing peptides (data not shown). Penicillin, ampicillin, erythromycin, and ceftazidime showed strong antibacterial activity against the *S. epidermidis* CICC 23664 strain, with MIC values of 0.78–1.56 μM, but weak activity against the clinically isolated MRSE 1208 strain, with MIC values of 50–100 μM. The MRSE 1208 strain was moderately susceptible to tetracycline, with an MIC value of 6.25 μM. These results are consistent with those of antimicrobial susceptibility testing (AST) performed by an automated MicroScan^®^ WalkAway 96 Plus system (Siemens Ltd., Germany).

**TABLE 2:** Antibacterial activity of peptides and antibiotics against the multiple-drug resistant *Staphylococcus epidermidis.*

	**MIC (μM)**								
	
	**Penicillin**	**Ampicillin**	**Ceftazidime**	**Erythromycin**	**Tetracycline**	**L11W**	**L12W**	**I1WL5W**	**I4WL5W**
MRSE1208	100	100	50	25	6.25	12.5	12.5	3.12	3.12
*S. epidermidis* (CICC 23664)	1.56	0.78	1.56	0.78	0.78	3.12	12.5	1.56	1.56

### Synergistic Antibacterial Activity of Antibiotics in Combination With Trp-Containing Peptides

**FIGURE 1:**
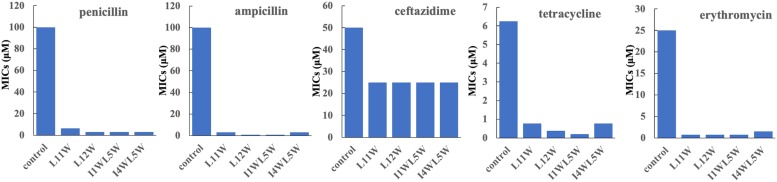
Antibacterial activity of antibiotics in combination with the Trp-containing peptides. Bacterial cultures were treated with a series of concentration of antibiotics in the presence of a low peptide concentration equivalent to one-fourth of its MIC at 37°C overnight. The OD600 was recorded using a microtiter plate reader. The MIC was defined as the lowest antibiotics concentration that inhibited 95% of the bacterial growth. Each data point represents an average of six independent experiments.

We chose five commercially available antibiotics, penicillin, ampicillin, erythromycin, ceftazidime, and tetracycline, to investigate whether the combination of the Trp-containing peptides with antibiotics provided a synergistic effect by using the checkerboard method. In combination with a low peptide concentration equivalent to one-fourth of its MIC, penicillin, ampicillin, erythromycin, and tetracycline had noticeably improved antimicrobial activity, with 32–64-fold reduced MIC values for ampicillin, 16–32-fold reduced MIC values for erythromycin and penicillin, and 8–16-fold reduced MIC values for tetracycline (Figure 1). The FICI that defines synergy between two antibacterial agents is shown in Table 3. FIC_a_ and FIC_b_ values are shown in the Supplementary Tables S2 and S3, respectively. The antibacterial activities of erythromycin, penicillin and ampicillin were synergistic with L11W (FICI values: 0.28–0.31), L12W (FICI values: 0.25–0.28), I1WL5W (FICI values: 0.25–0.28) and I4WL5W (FICI values: 0.15–0.31). Tetracycline showed synergistic effects with only I1WL5W (FICI value: 0.28) but additive effects with L11W, L12W and I4WL5W (FICI values: 0.56–0.62). Ceftazidime exhibited additive effects with L11W, L12W, I1WL5W, and I4WL5W (FICI values: 0.56–0.62).

**TABLE 3:** The fractional inhibitory concentration index (FICI) of the peptide/antibiotic combination against the MRSE 1208 strain.

	**FICI**				
	
	**Penicillin**	**Ampicillin**	**Ceftazidime**	**Erythromycin**	**Tetracycline**
L11W	0.3121	0.2808	0.6248	0.2808	0.6248
L12W	0.2808	0.2574	0.6248	0.2808	0.5624
I1WL5W	0.2812	0.2578	0.5641	0.2812	0.2820
I4WL5W	0.1875	0.1562	0.5641	0.3124	0.6248

*Synergy is defined as FICI ≤ 0.5, addition is defined as 0.5 < FICI ≤ 1.0, and indifference is defined as 1.0 < FICI ≤ 2.0 as indifference (Hollander et al., 1998; Yoon et al., 2004). Experiments were per formed in triplicate (*n* = 6).*

Our previous study showed that the Trp-containing peptides were low hemolysis (HC_50_ > 500 μM) (Bi et al., 2013, 2014). To estimate hemolytic and cytotoxic effect of the Trp-containing peptides in combination with the antibiotics, human erythrocytes and human epithelial cell 293T were used, respectively. The hemolytic activities of the peptides in combination with penicillin are as shown in Supplementary Figure S1A. one-fourth MIC L11W, L12W, I1WL5W or I4WL5W in combination with penicillin only caused less than 1% of hemolysis of erythrocytes. Similarly, the peptides in combination with penicillin showed approximately less than 15% of cytotoxicity (Supplementary Figure S1B).

### Synergistic Antibiofilm Activity of Common Antibiotics in Combination With Trp-Containing Peptides

**FIGURE 2:**
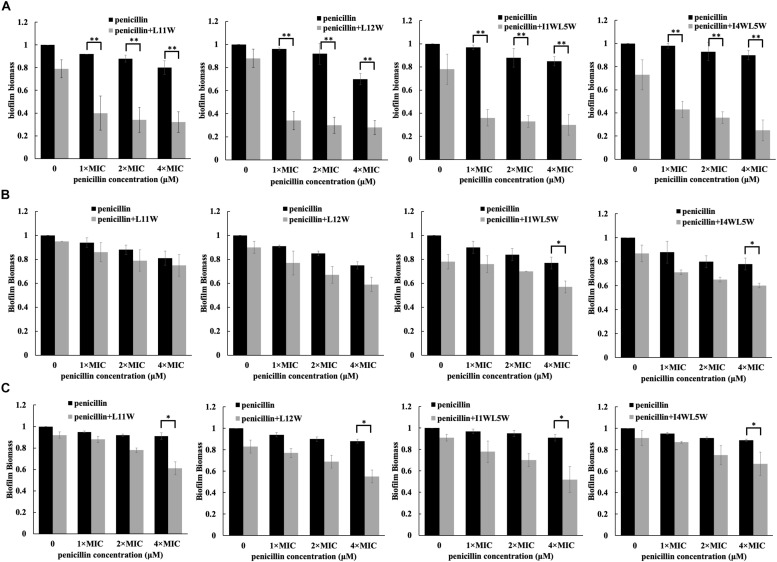
Effect of the combination of penicillin and peptides on the adhesion **(A)**, formation **(B)** and degradation **(C)** of biofilm. Log-phase bacteria were incubated with different concentrations of penicillin in combination with 1/4 × MIC of peptides at 37°C for 1 or 24 h, different concentration of penicillin as a single drug control and a concentration of penicillin 0 μM as a non-treated control. After removing the planktonic cells by centrifugation, the biofilms were washed, fixed and stained with crystal violet dye (CV). Biofilm biomass was quantified by using the following formula: OD590 of the sample/OD590 of the non-treated control (the black bar in a concentration of penicillin 0 μM). The biofilm biomass at 1 and 24 h represents the adhesion and formation of biofilm, respectively. The 24-h mature biofilm of MRSE 1208 cells was prepared as described in the Methods section, and then was treated with different concentrations of penicillin in combination with 1/4 × MIC of peptides at 37°C for 24 h, different concentration of penicillin as a single drug control and a concentration of penicillin 0 μM as a non-treated control. The biofilms were then fixed, stained and quantified as described above. Results represent the average and SEM of at least four independent experiments SEM (^∗^*P* < 0.05 and ^∗∗^*P* < 0.01).

In addition to a synergistic effect on the growth of planktonic MRSE 1208 cells, we found that the combination of antibiotics and the Trp-containing peptides showed an antibiofilm effect. As shown in Figure 2, 3.125, 6.25, and 12.5 μM penicillin alone were not able to affect biofilm formation, adhesion and degradation, but compared to penicillin alone, the combinations of penicillin with L11W, L12W, I1WL5W, and I4WL5W significantly decreased biofilm formation by 60–80% (Figure 2B), suggesting that the penicillin-peptide combination displayed highly synergistic activity on biofilm formation. However, the combination was less effective against biofilm adhesion (Figure 2A) and reduced 1-day-old MRSE 1208 biofilms only 10–20% at the 12.5 μM concentration of penicillin (Figure 2C). Similarly, ampicillin, erythromycin, tetracycline and ceftazidime exhibited antibiofilm activity in combination with the Trp-containing peptides against MRSE 1208 cells (Supplementary Figures S2–S5).

### Trp-Containing Peptide Inhibition of Gene Expression

**FIGURE 3:**
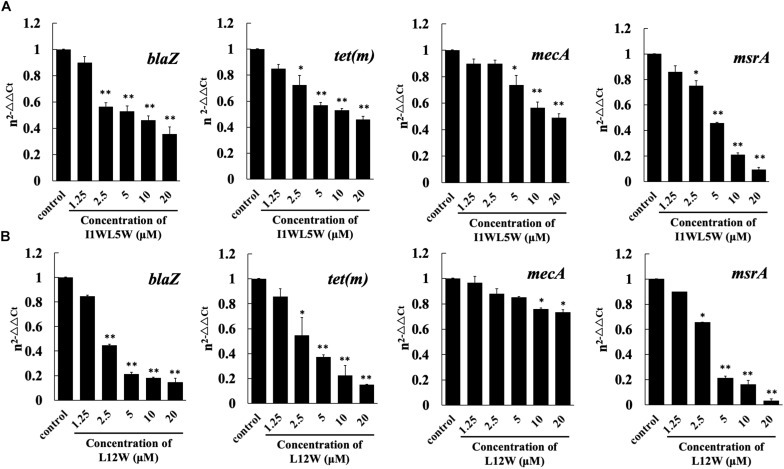
Effect of I1WL5W **(A)** and L12W **(B)** on expressions levels of the drug resistant-associated genes *bla*Z, *tet (m)*, *mec*A and *msr*A as assessed by real-time qPCR. The fold changes of genes were normalized to the housekeeping gene for GAPDH and further quantified relative to gene expression in MRSE1208 cells (control) was normalized to 1 using the comparative Ct method. Results represent the average and SEM of at least four independent experiments (^∗^*P* < 0.05 and ^∗∗^*P* < 0.01).

In Staphylococcus resistance, the *tet*(m) gene encodes a GTP-dependent Tet(M) protein that reduces the affinity of ribosomes for tetracycline when GTP is present and results in tetracycline resistance; *msr*A encodes an efflux protein that confers inducible resistance to macrolides, such as erythromycin (ERY); *mec*A or the recently discovered *bla*Z gene confer acquisition of β-lactamase and modification of normal penicillin-binding proteins (PBPs) that reflect different mechanisms of resistance for β-lactam antibiotics, such as penicillin, ceftazidime and ampicillin. To understand the effects of the Trp-containing peptides on specific resistance-associated proteins, we chose L12W and I1WL5W to test the expression level of relative genes using real-time qPCR (RT-qPCR). As shown in Figure 3, L12W significantly inhibited the expression of *bla*Z, *tet*(m) and *msr*A in a concentration-dependent manner. L12W at a concentration of 2.5 μM (≤1/4 × MIC) inhibited 55.3, 45.5, and 34.5% of the expression of *bla*Z, *tet*(m) and *msr*A, respectively, compared to the expression in the control condition (Figure 3A). Additionally, as the L12W concentration increased one-fold (5 μM), inhibition of expression of *bla*Z, *tet*(m) and *msr*A increased 78.7, 62.8, and 78.6% compared to the expression in the control condition, respectively. L12W at less than a concentration of 10 μM had no effect on the expression of *mec*A, and its MIC inhibited *mec*A expression by approximately 20%. I1WL5W at a concentration of 1.25 μM (1/4 × MIC = 0.78 μM) had no effect on the gene expression of *bla*Z, *mec*A, *tet*(m) and *msr*A. When its concentration increased one-fold (2.5 μM), the gene expression of *bla*Z, *tet*(m) and *msr*A was inhibited (Figure 3B).

### Membrane Activity of Trp-Containing Peptides

**FIGURE 4:**
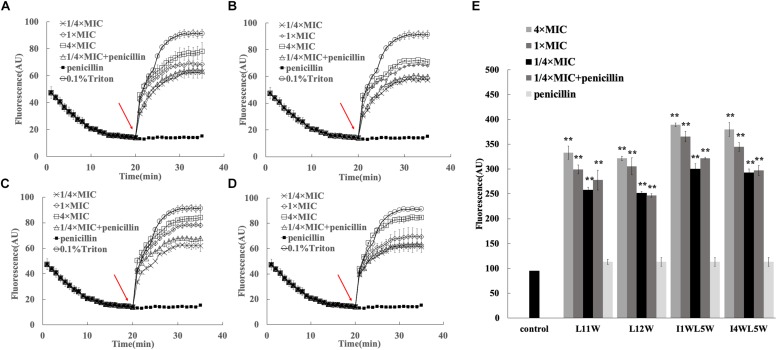
Effects of the combination of penicillin and peptides on membrane permeability. Plasma membrane depolarization of MRSE 1208 cells was measured in the presence of 1/4 MIC of L11W **(A)**, L12W **(B)**, I1WL5W **(C)** and I4WL5W **(D)** by using the membrane potential-sensitive fluorescence dye diSC3-5, as described in the Methods section. The arrow shows the time the peptide or penicillin was added. **(E)** Changes in outer membrane permeability induced by penicillin and peptides. Bacterial cells were incubated with NPN in the presence of various concentrations of the peptide or penicillin. The fluorescence intensity was measured when NPN was inserted into the hydrophobic interior of the outer membrane. Results represent the average and SEM of at least four independent experiments (^∗∗^*P* < 0.01).

Through comparison and analysis of the above results, we confirmed that the Trp-containing peptides at a 1/4 × MIC could be utilized to promote the antibacterial activity of antibiotics. Most AMPs can disturb membrane integrity to exert their bactericidal functions. To further elucidate the synergistic mechanism, we determined the membrane activity effects of peptides on the antibacterial activity of the antibiotic penicillin. Here, we investigated the effect of the Trp-containing peptides on the inner membrane by monitoring the depolarization level of the cell membrane of MRSE 1208 by using the potentiometric dye DiSC3-5, a membrane potential-sensitive dye. The fluorescence of DiSC3-5 becomes self-quenched as the dye enters the cytoplasm when the dye is added to bacteria. If the peptide induces depolarization of the inner membrane, DiSC3-5 will be released from the membrane, and the fluorescence will increase (Huang and Yousef, 2014). As shown in Figure 4, the Trp-containing peptides L11W (Figure 4A), L12W (Figure 4B), I1WL5W (Figure 4C) and I4WL5W (Figure 4D) induced cytoplasmic membrane depolarization in MRSE 1208 cells in a concentration-dependent manner, leading to an increase in DiSC3-5 fluorescence only after a lag time of 60 s. When the concentration of peptides increased to 4 × MIC, the level of depolarization was similar to that induced by Triton X-100, a representative membrane-rupturing compound, indicating that the Trp-containing peptides at a high concentration were capable of quickly permeating the inner membrane of MRSE 1208 cells and suggesting that this permeabilization is the mechanism by which these peptides kill bacteria. In contrast to the peptides, penicillin at a concentration of 3.125 μM did not influence the cytoplasmic membrane polarization of MRSE 1208 cells. The combination of 3.125 μM penicillin with 1/4 × MIC Trp-containing peptides induced similar levels of membrane depolarization to the treatment with 1/4 × MIC Trp-containing peptides alone, which could explain the antibacterial activity of 3.125 μM penicillin in the presence of the Trp-containing peptides.

The ability of the combination of the Trp-containing peptides and penicillin to permeabilize the outer membrane of MRSE 1208 cells was determined by a hydrophobic fluorescence probe, 1-N-phenylnapthylamine (NPN). NPN is unable to enter into an intact cell unless the outer membrane is disturbed by the addition of membrane-disrupting compounds, resulting in an increase in NPN fluorescence intensity (Mohanram and Bhattacharjya, 2014). Figure 4E shows that the Trp-containing peptides induced a significant increase in the fluorescence intensity of NPN in MRSE 1208 cells in a concentration-dependent manner. There were no significant changes in the fluorescence intensity of NPN upon the addition of penicillin compared to that of the control. The combination of penicillin and the Trp-containing peptides (1/4 × MIC) significantly damaged the permeability of the outer membranes in the MRSE 1208 cells and induced similar levels of membrane disruption to the treatment with 1/4 × MIC of the Trp-containing peptides. These results suggested that penicillin acted synergistically with the Trp-containing peptides possibly as a consequence of the peptide-mediated disruption of the outer and inner membrane integrity, which in turn efficiently enhanced the uptake of penicillin. Among these peptides, I1WL5W and I4WL5W at a concentration of 1/4 × MIC exhibited a stronger ability to induce outer membrane permeability than L11W and L12W.

**FIGURE 5:**
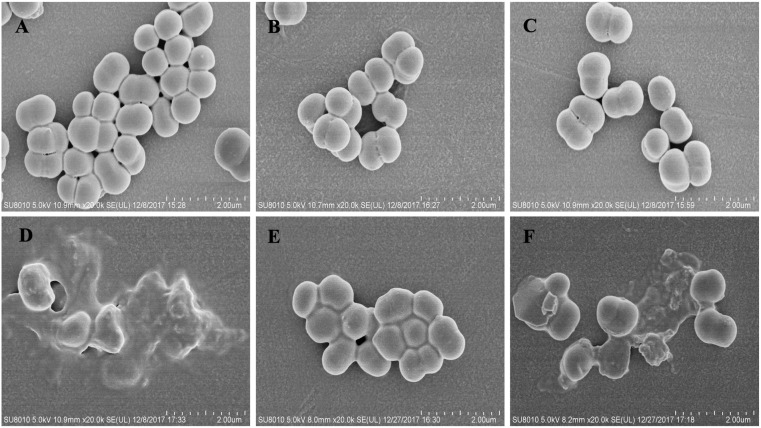
Scanning electron micrograph of MRSE 1208 cells. MRSE 1208 cells were treated with LB medium **(A)**, 3.125 μM penicillin **(B)**, 1/4 MIC of L12W **(C)** or 1/4 MIC of I1WL5W **(E)**, the combination of 3.125 μM penicillin and 1/4 MIC of L12W **(D)** or the combination of 3.125 μM penicillin and 1/4 MIC of I1WL5W **(F)** at 37°C for 1h. After washing, the bacterial cultures were fixed, dehydrated and coated with gold/palladium, and then observed under a scanning electron microscope, as described in the section “Materials and Methods”.

Representative SEM images of the MRSE 1208 strain following treatment with penicillin or peptide alone or both further confirmed the disruption of bacterial membranes (Figure 5). MRSE 1208 cells exhibited a normal shape and smooth surface. After treatment with 3.125 μM penicillin (Figure 5B) or with 1/4 × MIC of L12W (Figure 5C) alone, the overall morphology of the bacterial cells showed little change compared to that of a negative control group (Figure 5A). I1WL5W at a low concentration (1/4 MIC) slightly caused a bacterial cell surface disruption because of the increased permeability of inner and outer membrane, as shown in Figure 5E, the bacteria exhibited viscidity, which promoted aggregation. For the combination of penicillin and L12W (Figure 5D) or I1WL5W (Figure 5F), the integrity of the cell surface of MRSE 1208 was significantly damaged, resulting in cell lysis and intracellular content dispersal. The results above indicate that the peptides caused increased permeability and a loss of barrier function, which allows traditional antibiotics to enter the cytoplasm with ease and attack their cytoplasmic target in the combination of penicillin and peptides.

### Evaluating the Effects of Combinations of Antibiotics and Peptides in Infected Wounds

**FIGURE 6:**
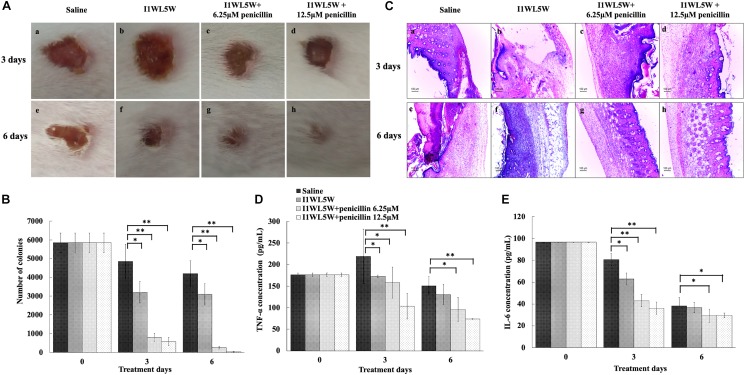
Synergistic effect of peptides and antibiotics against mouse wound infection caused by MRSE1208. **(A)** Representative macroscopic skin surface observation of a mouse after a 3-day and 6-day treatment following MRSE injection. A mouse model of a full-thickness skin infection was established. The wounds were treated with physiological saline (the negative control), I1WL5W (1/4 × MIC) alone, the combination of I1WL5W (1/4 × MIC)/6.25 μM penicillin or the combination of I1WL5W (1/4 × MIC)/12.5 μM penicillin every 12 h for 6 days. **(B)** The number of bacterial CFU in the wounded skin of mice treated with MRSE 1208 using the spiral-plating method. **(C)** Histological observation of a mouse skin biopsy stained by H&E after treatment of MRSE 1208. The pro-inflammatory factors TNF-α **(D)** and IL-6 **(E)**. Values represent the means ± SEMs (*n* = 8). ^∗^*P* < 0.05, ^∗∗^*P* < 0.01.

To further explore the synergistic effect of antibiotics and peptides *in vivo*, a mouse model of local wound infection with MRSE 1208 was established. A full-thickness skin section was excised, and then the wound was immediately infected with an MRSE 1208 culture (2 × 10^8^ CFU/ml). After 2 days of infection, different treatments were applied topically on the wound area every 12 h for 6 days. There were the following four groups: negative control (physiological saline), I1WL5W (1/4 × MIC), and the combinations of I1WL5W (1/4 × MIC) and 6.25 μM penicillin and I1WL5W (1/4 × MIC) and 12.5 μM penicillin. Figure 6A illustrates representative skin surface images of the mouse back after 3 and 6 days of treatment. A significant lesion was seen in the group of MRSE infections treated with physiological saline at 3 days of treatment. The severity of erythema and excoriation was similar for mice administered I1WL5W alone and the negative control. In contrast, the addition of I1WL5W and 6.25 μM penicillin or I1WL5W and 12.5 μM penicillin reduced the abscess caused by MRSE infection; the wounds were smaller than they were originally and were pink in color. After 6 days of treatment, an improvement in lesion healing was observed in the mouse skin that was administered I1WL5W alone, and the lesion of the wound was significantly reduced. It was surprising that nearly complete lesion healing on the infected skin surface was observed in mice treated with the combination of I1WL5W and 6.25 μM penicillin or I1WL5W and 12.5 μM penicillin. No sign of inflammation could be observed, and the lesions started to heal, with new hair growing around them.

The number of bacterial CFU in the wounded skin tissue was determined (Figure 6B). At 3 days of treatment, the number of bacterial CFU in the wounds treated with physiological saline and with I1WL5W alone remained approximately 4850 and 3200 CFU/g tissue, respectively. The combination of I1WL5W and 6.25 μM penicillin or I1WL5W and 12.5 μM penicillin significantly decreased the bacterial counts to 800 and 580 CFU/g, respectively. At day 6, the number of bacterial CFU was not significantly reduced in the mice treated with physiological saline and I1WL5W alone when compared to that at day 3. However, the combination of I1WL5W and 6.25 μM penicillin or I1WL5W and 12.5 μM penicillin resulted in a 95–99% reduction compared with the physiological saline treatment, with bacterial counts of 250 and 35 CFU/g, respectively.

Furthermore, skin histological morphology observations confirmed the synergistic effects of antibiotic and peptides on the infection of MRSE (Figure 6C). At day 3, the skin treated with physiological saline and I1WL5W alone showed a significant disorganization in the epidermis and degenerated dermis, and immune cell infiltration was observed in the subcutaneous region, suggesting deep inflammation. After treatment with a combination of I1WL5W and 6.25 μM penicillin or I1WL5W and 12.5 μM penicillin, neutrophil accumulation in the MRSE-infected skin was found to be attenuated. The combination of I1WL5W and penicillin displayed greater amelioration at day 6 than that at day 3 of treatment.

To better understand the protective activity of the antibiotic and peptide combination against infection with MRSE, the levels of the proinflammatory cytokines TNF-α and IL-6 in serum were determined. MRSE induced a systemic response of proinflammatory cytokines, resulting in a significant increase in the levels of the proinflammatory cytokines TNF-α (Figure 6D) and IL-6 (Figure 6E) in serum after physiological saline administration. The MRSE 1208-treated mice exhibited decreased serum cytokine levels when further treated with a combination of I1WL5W and penicillin. The combination of I1WL5W and 12.5 μM penicillin was able to inhibit TNF-α and IL-6 expression by 96.8 and 95.6%, respectively.

## Discussion

Inappropriate antibiotic use leads to the emergence of MRSE strains, which increase treatment difficulty and complexity. AMPs are considered to be a novel class of potent antimicrobial agents because they have broad-spectrum antimicrobial activity and exclusive membrane action mechanisms and are less likely than current antibiotics to induce drug resistance (Zasloff, 2002; Brogden, 2005; Niyonsaba et al., 2010). Previous studies have demonstrated a synergistic effect of some synthetic short and cationic AMPs in combination with conventional antibiotics (Maisetta et al., 2009; Kajal et al., 2015; Regmi et al., 2017a). Synergy reduces the MIC of the combined antibiotic and peptides and helps prevent the development of resistance in microorganisms (Wu et al., 1999; Regmi et al., 2017b). The generic membrane-disrupting activity of AMPs increases access to the cell for conventional small molecule antibiotics, including ceftriaxone, amoxicillin clavulanate, ceftazidime, meropenem, piperacillin and β-lactam antibiotics, and exerts synergistic effects (Molinari et al., 1993; Falla and Hancock, 1997; Zhou and Peng, 2013). The present study confirmed the synergistic effect between the Trp-containing peptides L11W, L12W, I1WL5W, and I4WL5W and the commonly used antibiotics penicillin, ampicillin, erythromycin, ceftazidime and tetracycline against the clinically isolated MRSE 1208 strain. When used as monotherapy, penicillin, ampicillin and erythromycin showed weak antibacterial activity against the clinically isolated MRSE 1208 strain, with MIC values of 50–100 μM, but strong activity against the *S. epidermidis* CICC 23664 strain, with MIC values of 0.78–1.56 μM. However, their antibacterial activities were significantly improved in combination with the Trp-containing peptides at a low concentration equivalent to one-fourth of the MICs, with 32–64-fold reduced MIC values for ampicillin and 16–32-fold reduced MIC values for erythromycin and penicillin. In addition, a synergistic effect was also observed for the combination of the peptides and penicillin, ampicillin or erythromycin against MRSE 1208 strain by using checkerboard assays. Tetracycline showed synergistic activity with only I1WL5W but additive activity with L11W, L12W, and I4WL5W. Ceftazidime exhibited additive activity with the four Trp-containing peptides. Similarly, Zhou et al. demonstrated synergies between ranalexin and polymyxin E, doxycycline and clarithromycin, and magainin II and ceftriaxone, amoxicillin clavulanate, ceftazidime, meropenem, piperacillin and β-lactam antibiotics (Zhou and Peng, 2013). Ceftazidime was not observed to be synergistic with indolicidin, a Trp-containing peptide, against methicillin-resistant *S. aureus* (MRSA), although it was demonstrated to be synergistic with Esc (1–18), magainin II, polymyxin E and ranalexin against *Stenotrophomonas maltophilia* and MRSA in previous studies (Maisetta et al., 2009; Zhou and Peng, 2013). Our results suggest that the Trp-containing peptides may be used as promising synergistic agents to improve the antibacterial effectiveness of the selected antibiotics against MRSE and to reduce the therapeutic dose of antibiotics, thus minimizing their toxic side effects.

Conventional antibiotics are less effective in treating biofilm bacteria than in treating planktonic bacteria because biofilms coated with an external polysaccharide matrix display increased resistance to antibiotic delivery and immune system clearance (Donlan and Costerton, 2002; Høiby et al., 2010; Nagant et al., 2012). As expected, we found that the tested five conventional antibiotics had no effect on the formation of biofilms by the MRSE 1208 strain. However, combination of these antibiotics with the Trp-containing peptides proved to be highly successful in inhibiting biofilm formation. Similarly, Almaaytah et al. demonstrated that the combinations of ampicillin and erythromycin with MelitAP-27, a novel hybrid antimicrobial peptide, displayed highly significant synergistic activity against biofilm formation by *S. aureus* (33591) and *Pseudomonas aeruginosa* (BAA2114), respectively (Almaaytah et al., 2016). FLIP7 is an AMP complex of the blowfly *Calliphora vicina* containing a combination of defensins, cecropins, diptericins and proline-rich peptides and has also demonstrated high synergistic antibiofilm activity with ampicillin and erythromycin against *S. aureus* and *Escherichia coli* (Chernysh et al., 2018). The synergistic effect allowed reduction of the effective antibiofilm concentration of the antibiotic to a level well below the one clinically achievable. In the present study, the tested antibiotics, representing a range of different antibiotic classes, had different antibacterial targets and pathways; however, they exhibited synergistic activities against biofilm formation when they were combined with the Trp-containing peptides, suggesting that the peptides induce bacterial death and inhibition through a mechanism of action different from all of the mechanisms of the investigated antibiotics.

It is generally known that most cationic AMPs induce cell death through the ability to disrupt membrane structure and generate pores (Radovic-Moreno et al., 2012). In Trp-containing peptides, tryptophan residues are able to deeply insert into negatively charged cell membranes, resulting in strong membrane-disruptive activity, and this property endows Trp-containing AMPs with a unique ability to interact with the surface of bacterial cell membranes (Schiffer et al., 1992; Rex, 2000; Bi et al., 2013, 2014). In this study, we tested the hypothesis that membrane disruption will lead to generic synergistic interactions between Trp-containing peptides and chemical antibiotics. Depolarization of the bacterial plasma membrane provides a direct assessment of the effects on membrane permeability. The four Trp-containing peptides exhibited the ability to quickly permeate the inner membrane of MRSE 1208 cells, suggesting that these peptides kill bacteria. As expected, penicillin did not influence the cytoplasmic membrane polarization of MRSE 1208 cells, but the combination of penicillin with 1/4 × MIC Trp-containing peptides induced membrane depolarization. Notably, compared to the treatment with the Trp-containing peptides alone, the combination treatment of penicillin and peptide did not significantly increase the membrane-disruptive activity, suggesting that the synergistic bactericidal effect observed in the combination of peptide and penicillin against the MRSE 1208 strain may result from the membrane-disruptive activity of Trp-containing peptides leading to enhanced intracellular uptake of antibiotics to rapidly and efficiently kill bacteria. The bacterial outer membrane is a barrier to the uptake of antibiotics due to the presence of lipopolysaccharides and teichoic acid (Mohanram and Bhattacharjya, 2014; Hemeg, 2017). The Trp-containing peptides significantly increased the permeability of the outer membrane of MRSE 1208 cells in a concentration-dependent manner. I1WL5W and I4WL5W at a concentration of 1/4 × MIC exhibited a stronger outer membrane-permeabilizing activity than L11W and L12W. There were no significant changes in the fluorescence intensity of NPN upon the addition of penicillin alone, and the penicillin and peptide combination increased the permeability of the outer membrane of the MRSE 1208 cells but displayed no difference from that for the Trp-containing peptides alone. The results suggest that penicillin acted synergistically with the Trp-containing peptides possibly as a consequence of the peptide-mediated disruption of the outer and inner membrane integrity, which in turn efficiently enhanced the uptake of penicillin into bacterial cells to generate a high local drug concentration to kill bacteria. SEM images further confirmed the disruption of the bacterial membranes of the MRSE 1208 strain following treatment with the combination of penicillin and peptide. The above results further demonstrate the mechanisms underlying the synergistic behavior of the AMP-antibiotic combinations proposed by Almaaytah et al. (2016) as the membrane-disruptive ability of AMPs could assist antibiotics in reaching their molecular targets to rapidly and efficiently kill bacteria.

**FIGURE 7:**
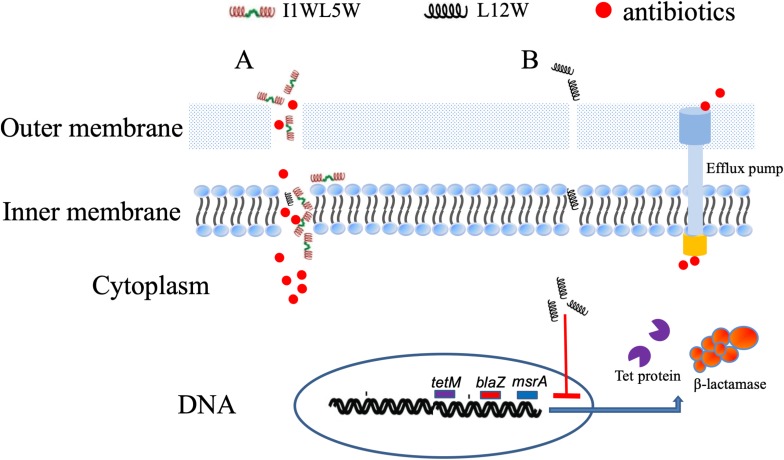
The possible mechanisms of the antibiotics/Trp-containing peptides combination for MRSE killing. **(A)** The peptides containing two Trp-residues (I1WL5W) increase the permeability of the outer and inner membrane of MRSE 1208 cells, which efficiently enhances the uptake of antibiotics into bacterial cells to generate a high local drug concentration and kill bacteria. **(B)** The peptides containing one Trp-residue (L12W) may increase intracellular antibiotic levels by decreasing *bla*Z, *tet*(m) and *msr*A expression in addition to disrupting the outer membrane of MRSE 1208 cells.

In Staphylococcus resistance, *mec*A or the recently discovered *bla*Z gene confer the acquisition of β-lactamase and modification of normal PBPs that reflect different mechanisms of resistance for β-lactam antibiotics, and the *tet*(m) and *msr*A genes encode a GTP-dependent Tet(M) protein and an efflux protein, conferring inducible resistance to tetracycline and macrolides, respectively (Spiegel, 1988; Bliziotis et al., 2005; Fernandes, 2006; Feng et al., 2015). L12W at a concentration of 2.5 μM, which is lower than the concentration of L12W in combination with penicillin (3.12 μM), significantly downregulated the expression of *bla*Z, *tet*(m) and *msr*A, which can lead to inhibition of protein translation of PBPs, the GTP-dependent Tet(M) protein and the efflux protein to reduce the resistance of MRSE 1208 to penicillin, ampicillin, erythromycin, and tetracycline. However, I1WL5W at a concentration of 1.25 μM, which is more than the concentration of I1WL5W in combination with penicillin (0.78 μM), showed no effect on the level of transcription of the three genes. These results suggest that L12W may increase intracellular antibiotic levels by decreasing *bla*Z, *tet*(m) and *msr*A expression in addition to disrupting the outer membrane of MRSE 1208 cells but that I1WL5W function by only disrupting the outer membrane of MRSE 1208 cells. The possible mechanisms by which the combination of an antibiotic and a Trp-containing peptide promotes MRSE killing are illustrated in Figure 7. As shown in the results above, penicillin, ampicillin, and erythromycin exhibited strong synergistic effects in combination with the Trp-containing peptides against MRSE 1208 *in vitro*, and their antibacterial activities were significantly improved. *In vivo*, similar results were obtained for the I1WL5W and penicillin combination against a mouse wound infection caused by the MRSE 1208 strain. In our mouse wound infection study, the I1WL5W and penicillin combination was able to significantly lessen the abscess caused by MRSE infection, and nearly complete lesion healing of the infected skin surface was observed. The combination of I1WL5W and penicillin resulted in a 95–99% reduction in CFU, and cytokine TNF-α and IL-6 levels in serum were decreased 96.8 and 95.6%, respectively.

## Conclusion

Our study reveals the synergistic activity of five commercially available antibiotics in combination with Trp-containing peptides against MRSE *in vitro* and *in vivo*. This combination therapy allows a lower dose of traditional antibiotics to be used while maintaining antibacterial activities, which is helpful for delaying the emergence of resistance. Bacterial inner and outer membranes are the main targets of Trp-containing peptides, especially I1WL5W and I4WL5W, both with 2 Trp residues. Peptides cause increased permeability and a loss of barrier function, which allows traditional antibiotics to enter the cytoplasm with ease and attack their cytoplasmic target. Importantly, our study provides a potential therapeutic option for antibiotic-resistant pathogens by combining AMPs and a range of different traditional antibiotic classes.

## Data Availability Statement

All datasets generated for this study are included in the article/Supplementary Material.

## Ethics Statement

This animal experiment was carried out in accordance with the recommendations of the Animal Welfare and Research Ethics Committee of Liaoning Normal University. This study uses strains obtained from a clinical specimen submitted to the First Affiliated Hospital of Dalian Medical University. The Ethics Committee of the First Affiliated Hospital of Dalian Medical University did not require the study to be reviewed or approved by an ethics committee because the strain isolated for clinical diagnosis was used in scientific research, rather than for scientific research.

## Author Contributions

DS conceived and designed the experiments and wrote the manuscript. YL, FeJ, and XH performed the experiments. FaJ performed the determination of the antibacterial activity of the peptides and antibiotics against the *Staphylococcus epidermidis* (CICC 23664) strain, and hemolysis of the peptides and antibiotics against human erythrocytes. HW performed the determination of cytotoxicity of the peptides and antibiotics for human kidney 293T cells.

## Conflict of Interest

The authors declare that the research was conducted in the absence of any commercial or financial relationships that could be construed as a potential conflict of interest.

## Supplementary Material

FIGURE S1 Cytotoxicity of the peptides in combination with penicillin. **(A)** hemolysis on human erythrocyte; **(B)** Cell viability of human renal epithelial cell 293T.

FIGURE S2 Effect of the combination of ampicillin and peptides on the adhesion **(A)**, formation **(B)** and degradation **(C)** of biofilm. Log-phase bacteria were incubated with different concentrations of ampicillin in combination with 1/4 × MIC of peptides at 37°C for 1 or 24 h, different concentration of ampicillin as a single drug control and a concentration of ampicillin 0 μM as a nontreated control. After removing the planktonic cells by centrifugation, the biofilms were washed, fixed and stained with crystal violet dye (CV). Biofilm biomass was quantified by using the following formula: OD590 of the sample/OD590 of the nontreated control (the black bar in a concentration of ampicillin 0 μM). The biofilm biomass at 1 and 24 h represents the adhesion and formation of biofilm, respectively. The 24-h mature biofilm of MRSE 1208 cells was prepared as described in the Methods section, and then was treated with different concentrations of ampicillin in combination with 1/4 × MIC of peptides at 37°C for 24 h, different concentration of ampicillin as a single drug control and a concentration of ampicillin 0 μM as a nontreated control. The biofilms were then fixed, stained and quantified as described above. Results represent the average and SEM of at least four independent experiments SEM (^∗^*P* < 0.05 and ^∗∗^*P* < 0.01).

FIGURE S3 Effect of the combination of erythromycin and peptides on the adhesion **(A)**, formation **(B)** and degradation **(C)** of biofilm. Log-phase bacteria were incubated with different concentrations of erythromycin in combination with 1/4 × MIC of peptides at 37°C for 1 or 24 h, different concentration of am erythromycin as a single drug control and a concentration of erythromycin 0 μM as a nontreated control. After removing the planktonic cells by centrifugation, the biofilms were washed, fixed and stained with crystal violet dye (CV). Biofilm biomass was quantified by using the following formula: OD590 of the sample/OD590 of the nontreated control (the black bar in a concentration of erythromycin 0 μM). The biofilm biomass at 1 and 24 h represents the adhesion and formation of biofilm, respectively. The 24-h mature biofilm of MRSE 1208 cells was prepared as described in the Methods section, and then was treated with different concentrations of erythromycin in combination with 1/4 × MIC of peptides at 37°C for 24 h, different concentration of erythromycin as a single drug control and a concentration of erythromycin 0 μM as a nontreated control. The biofilms were then fixed, stained and quantified as described above. Results represent the average and SEM of at least four independent experiments SEM (^∗^*P* < 0.05 and ^∗∗^*P* < 0.01).

FIGURE S4 Effect of the combination of tetracycline and peptides on the adhesion **(A)**, formation **(B)** and degradation **(C)** of biofilm. Log-phase bacteria were incubated with different concentrations of tetracycline in combination with 1/4 × MIC of peptides at 37°C for 1 or 24 h, different concentration of tetracycline as a single drug control and a concentration of tetracycline 0 μM as a nontreated control. After removing the planktonic cells by centrifugation, the biofilms were washed, fixed and stained with crystal violet dye (CV). Biofilm biomass was quantified by using the following formula: OD590 of the sample/OD590 of the nontreated control (the black bar in a concentration of tetracycline 0 μM). The biofilm biomass at 1 and 24 h represents the adhesion and formation of biofilm, respectively. The 24-h mature biofilm of MRSE 1208 cells was prepared as described in the Methods section, and then was treated with different concentrations of tetracycline in combination with 1/4 × MIC of peptides at 37°C for 24 h, different concentration of tetracycline as a single drug control and a concentration of tetracycline 0 μM as a nontreated control. The biofilms were then fixed, stained and quantified as described above. Results represent the average and SEM of at least four independent experiments SEM (^∗^*P* < 0.05 and ^∗∗^*P* < 0.01).

FIGURE S5 Effect of the combination of ceftazidime and peptides on the adhesion **(A)**, formation **(B)** and degradation **(C)** of biofilm. Log-phase bacteria were incubated with different concentrations of ceftazidime in combination with 1/4 × MIC of peptides at 37°C for 1 or 24 h, different concentration of ceftazidime as a single drug control and a concentration of ceftazidime 0 μM as a nontreated control. After removing the planktonic cells by centrifugation, the biofilms were washed, fixed and stained with crystal violet dye (CV). Biofilm biomass was quantified by using the following formula: OD590 of the sample/OD590 of the nontreated control (the black bar in a concentration of ceftazidime 0 μM). The biofilm biomass at 1 and 24 h represents the adhesion and formation of biofilm, respectively. The 24-h mature biofilm of MRSE 1208 cells was prepared as described in the Methods section, and then was treated with different concentrations of ceftazidime in combination with 1/4 × MIC of peptides at 37°C for 24 h, different concentration of ceftazidime as a single drug control and a concentration of ceftazidime 0 μM as a nontreated control. The biofilms were then fixed, stained and quantified as described above. Results represent the average and SEM of at least four independent experiments SEM (^∗^*P* < 0.05 and ^∗∗^*P* < 0.01.

TABLE S1 The sequence of primer in this study.

TABLE S2 The fractional inhibitory concentration of the peptides in the combination and the antibiotics (FIC_a_).

TABLE S3 The fractional inhibitory concentration of the antibiotics in the combination and the peptides (FIC_b_).

## References

[B1] Abd-El-Aziz A. S. Agatemor C. Etkin N. Overy D. P. Lanteigne M. McQuillan K. (2015). Antimicrobial organometallic dendrimers with tunable activity against multidrug-resistant bacteria. *Biomacromolecules* 16 3694–3703. 10.1021/acs.biomac.5b01207 26452022

[B2] Almaaytah A. Alnaamneh A. Abualhaijaa A. Alshari N. Al-Balas Q. (2016). In vitro synergistic activities of the hybrid antimicrobial peptide melitap-27 in combination with conventional antibiotics against planktonic and biofilm forming bacteria. *Int. J. Pept. Res. Ther.* 22 497–504. 10.1007/s10989-016-9530-z

[B3] Ashburn T. T. Thor K. B. (2004). Drug repositioning: identifying and developing new uses for existing drugs. *Nat. Rev. Drug Discov.* 3 673–683. 10.1038/nrd1468 15286734

[B4] Berditsch M. Jäger T. Strempel N. Schwartz T. Overhage J. Ulricha A. S. (2015). Synergistic effect of membrane-active peptides polymyxin b and gramicidin son multidrug-resistant strains and biofilms of *Pseudomonas aeruginosa*. *Antimicrob. Agents Chemother.* 59 5288–5296. 10.1128/aac.00682-15 26077259 PMC4538509

[B5] Bi X. Wang C. Dong W. Zhu W. Shang D. (2014). Antimicrobial properties and interaction of two Trp-substituted cationic antimicrobial peptides with a lipid bilayer. *J. Antibiot.* 67 361–368. 10.1038/ja.2014.4 24496141

[B6] Bi X. Wang C. Ma L. Sun Y. Shang D. (2013). Investigation of the role of tryptophan residues in cationic antimicrobial peptides to determine the mechanism of antimicrobial action. *J. Appl. Microbiol.* 115 663–672. 10.1111/jam.12262 23710779

[B7] Bliziotis I. A. Samonis G. Vardakas K. Z. Chrysanthopoulou S. Falagas M. E. (2005). Effect of aminoglycoside and beta-lactam combination therapy versus beta-lactam monotherapy on the emergence of antimicrobial resistance: a meta-analysis of randomized, controlled trials. *Clin. Infect. Dis.* 41 149–158. 10.1086/430912 15983909

[B8] Bracho D. O. Barsan L. Arekapudi S. R. Thompson J. A. Hen J. Stern S. A. (2009). Antibacterial properties of an iron−based hemostatic agent in vitro and in a rat wound model. *Acad Emerg Med.* 16 656–660. 10.1111/j.1553-2712.2009.00439.x 19538502

[B9] Brogden K. A. (2005). Antimicrobial peptides: pore formers or metabolic inhibitors in bacteria? *Nat. Rev. Microbiol.* 3 238–250. 15703760 10.1038/nrmicro1098

[B10] Carver T. Bleasby A. (2003). The design of Jemboss: a graphical user interface to EMBOSS. *Bioinformatics* 19 1837–1843. 10.1093/bioinformatics/btg251 14512356

[B11] Chernysh S. Gordya N. Tulin D. Yakovlev A. (2018). Biofilm infections between scylla and charybdis: interplay of host antimicrobial peptides and antibiotics. *Infect. Drug Resist.* 11 501–514. 10.2147/IDR.S157847 29674848 PMC5898886

[B12] Dong W. Zhu X. Yang Y. Yan X. Sun L. Shang D. J. (2018). Potential role of a series of lysine/leucine-rich antimicrobial peptide in inhibiting lipopolysaccharide-induced inflammation. *Biochemical. J.* 475 3687–3706. 10.1042/BCJ20180483 30373763

[B13] Donlan R. M. Costerton J. W. (2002). Biofilms: survival mechanisms of clinically relevant microorganisms. *Clin. Microbiol. Rev.* 15 167–193. 10.1128/cmr.15.2.167-193.2002 11932229 PMC118068

[B14] Eisenberg D. Weiss R. M. Terwilliger T. C. (1982). The helical hydrophobic moment: a measure of the amphiphilicity of a helix. *Nature* 299 371–374. 7110359 10.1038/299371a0

[B15] Falla T. Hancock R. E. (1997). Improved activity of a synthetic indolicidin analog. *Antimicrob. Agents Chemother.* 41 771–775. 10.1128/aac.41.4.771 9087487 PMC163792

[B16] Feng Q. Huang Y. Chen M. Li G. Chen Y. (2015). Functional synergy of α-helical antimicrobial peptides and traditional antibiotics against Gram-negative and Gram-positive bacteria in vitro and in vivo. *Eur. J. Clin. Microbiol. Infect. Dis.* 34 197–204. 10.1007/s10096-014-2219-3 25169965

[B17] Fernandes P. (2006). Antibacterial discovery and development—the failure of success? *Nat. Biotechnol.* 24 1497–1503. 10.1097/01.ASW.0000456631.20389.ae 17160049

[B18] Fjell C. D. Hiss J. A. Hancock R. E. W. Schneider G. (2011). Designing antimicrobial peptides: from follows function. *Nat. Rev. Drug Discov.* 11 37–51. 10.1038/nrd3591 22173434

[B19] Hemeg H. A. (2017). Nanomaterials for alternative antibacterial therapy. *Int. J. Nanomed.* 12 8211–8225. 10.2147/IJN.S132163 29184409 PMC5689025

[B20] Høiby N. Bjarnsholt T. Givskov M. Molin S. Ciofu O. (2010). Antibiotic resistance of bacterial biofilms. *Int. J. Antimicrob. Agents* 35 322–332. 10.1016/j.ijantimicag.2009.12.011 20149602

[B21] Hollander J. G. Mouton J. W. Verbrugh H. A. (1998). Use of pharmacodynamic parameters to predict efficacy of combination therapy by using fractional inhibitory concentration kinetics. *Antimicrob. Agents Chemother.* 42 744–748. 10.1128/aac.42.4.744 9559776 PMC105535

[B22] Huang E. Yousef A. E. (2014). Lipopeptide antibiotic, paenibacterin, binds to bacterial outer membrane and exerts bactericidal activity through cytoplasmic membrane damage. *Appl. Environ. Microbiol.* 80 2700–2704. 10.1128/AEM.03775-13 24561581 PMC3993306

[B23] Kajal G. Singh S. Monique L. H. (2015). Short, synthetic cationic peptides have antibacterial activity against mycobacterium smegmatis by forming pores in membrane and synergizing with antibiotics. *Antibiotics* 4 358–378. 10.3390/antibiotics4030358 27025629 PMC4790291

[B24] Khandelia H. Kaznessis Y. N. (2007). Cation–π interactions stabilize the structure of the antimicrobial peptide indolicidin near membranes: molecular dynamics simulations. *J. Phys. Chem. B.* 111 242–250. 10.1021/jp064776j 17201448 PMC2440664

[B25] Khara J. S. Wang Y. Ke X. Y. Liu S. Newton S. M. Langford P. R. (2014). Anti-mycobacterial activities of synthetic cationic alpha-helical peptides and their synergism with rifampicin. *Biomaterials* 35 2032–2038. 10.1016/j.biomaterials.2013.11.035 24314557

[B26] Maisetta G. Mangoni M. L. Esin S. Pichierri G. Capria A. L. Brancatisano F. L. (2009). In vitro bactericidal activity of the N-terminal fragment of the frog peptide esculentin-1b (Esc 1-18) in combination with conventional antibiotics against *Stenotrophomonas* maltophilia. *Peptides* 30 1622–1626. 10.1016/j.peptides.2009.06.004 19520127

[B27] Mant C. T. Kovacs J. M. Kim H. M. Pollock D. D. Hodges R. S. (2009). Intrinsic amino acid side-chain hydrophilicity/hydrophobicity coefficients determined by reversed-phase high-performance liquid chromatography of model peptides: comparison with other hydrophilicity/hydrophobicity scales. *Biopolymers* 92 573–595. 10.1002/bip.21316 19795449 PMC2792893

[B28] Marr A. K. Gooderham W. J. Hancock R. E. (2006). Antibacterial peptides for therapeutic use: obstacles and realistic outlook. *Curr. Opin. Pharmacol.* 6 468–472. 10.1016/j.coph.2006.04.006 16890021

[B29] Mohanram H. Bhattacharjya S. (2014). Resurrecting inactive antimicrobial peptides from the lipopolysaccharide trap. *Antimicrob. Agents Chemother.* 58 1987–1996. 10.1128/AAC.02321-13 24419338 PMC4023739

[B30] Molinari G. Guzmaìn C. A. Pesce A. Schito G. C. (1993). Inhibition of *Pseudomonas aeruginosa* virulence factors by subinhibitory concentrations of azithromycin and other macrolide antibiotics. *J. Antimicrob. Chemother.* 31 681–688. 10.1093/jac/31.5.681 8392997

[B31] Nagant C. Pitts B. Nazmi K. Vandenbranden M. Bolscher J. G. Stewart P. S. (2012). Identification of peptides derived from the human antimicrobial peptide LL-37 active against biofilms formed by *Pseudomonas aeruginosa* using a library of truncated fragments. *Antimicrob. Agents Chemother.* 56 5698–5708. 10.1128/AAC.00918-12 22908164 PMC3486595

[B32] Niyonsaba F. Ushio H. Hara M. Yokoi H. Tominaga M. Takamori K. (2010). Antimicrobial peptides human beta-defensins and cathelicidin LL-37 induce the secretion of a pruritogenic cytokine IL-31 by human mast cells. *J. Immunol.* 184 3526–3534. 10.4049/jimmunol.0900712 20190140

[B33] Otto M. (2014). Staphylococcus epidermidis pathogenesis. *Methods Mol. Biol.* 1106 17–31. 10.1007/978-1-62703-736-5_2 24222452

[B34] Pasupuleti M. Malmsten M. Schmidtchen A. (2011). Antimicrobial peptides: a key component of innate immunity. *Crit. Rev. Biotechnol.* 32 143–171. 22074402 10.3109/07388551.2011.594423

[B35] Radovic-Moreno A. F. Lu T. K. Puscasu V. A. Yoon C. J. Langer R. Farokhzad O. C. (2012). surface charge-switching polymeric nanoparticles for bacterial cell wall-targeted delivery of antibiotics. *ACS Nano* 6 4279–4287. 10.1021/nn3008383 22471841 PMC3779925

[B36] Regmi S. Choi Y. H. Choi Y. S. Kim M. R. Yoo J. C. (2017a). Antimicrobial peptide isolated from Bacillus amyloliquefaciens K14 revitalizes its use in combinatorial drug therapy. *Folia Microbiol.* 62 127–138. 10.1007/s12223-016-0479-2 27787755

[B37] Regmi S. Yoon S. C. Yun H. C. Young K. K. Seung S. C. Jin C. Y. (2017b). Antimicrobial peptide from *Bacillus* subtilis CSB138: characterization, killing kinetics, and synergistic potency. *Int. Microbiol.* 20 43–53. 10.2436/20.1501.01.284 28581021

[B38] Rex S. (2000). A Pro-Ala substitution in melittin affects self-association, membrane binding and pore-formation kinetics due to changes in structural and electrostatic properties. *Biophys. Chem.* 85 209–228. 10.1016/s0301-4622(00)00121-6 10961508

[B39] Schiffer M. Chang C. H. Stevens F. J. (1992). The functions of tryptophan residues in membrane proteins. *Protein Eng.* 5 213–214. 10.1093/protein/5.3.213 1409540

[B40] Shang D. Li X. Sun Y. Wang C. Sun L. Wei S. (2012). Design of potent, non-toxic antimicrobial agents based upon the structure of the frog skin peptide, temporin-1CEb from Chinese brown frog. *Rana Chensinensis*. *Chem. Biol. Drug Des.* 79 653–662. 10.1111/j.1747-0285.2012.01363.x 22348663

[B41] Shang D. Yu F. Li J. Zheng J. Zhang L. Li Y. (2009). Molecular cloning of cDNAs encoding antimicrobial peptide precursors from the skin of the Chinese brown frog Rana chensinensis. *Zoolog. Sci.* 26 220–226. 10.2108/zsj.26.220 19341344

[B42] Shang D. J. Liang H. Wei S. Yan X. Yang Q. Sun Y. (2014). Effects of antimicrobial peptide L-K6, a temporin-1CEb analog on oral pathogen growth, *Streptococcus mutans* biofilm formation, and anti-inflammatory activity. *Appl. Microbio. Biotech.* 98 8685–8695. 10.1007/s00253-014-5927-9 25056289

[B43] Soren O. Brinch K. S. Patel D. Liu Y. Liu A. Coates A. (2015). Antimicrobial peptide novicidin synergizes with rifampin, ceftriaxone, and ceftazidime against antibiotic-resistant *Enterobacteriaceae* in vitro. *Antimicrob. Agents Chemother..* 59 6233–6240. 10.1128/AAC.01245-15 26248380 PMC4576052

[B44] Spellberg B. Bartlett J. G. Gilbert D. N. (2013). The future of antibiotics and resistance. *N. Engl. J. Med.* 368 299–302. 23343059 10.1056/NEJMp1215093 PMC3617123

[B45] Spiegel C. A. (1988). Laboratory detection of high-level aminoglycoside-aminocyclitol resistance in *Enterococcus* spp. *J. Clin. Microbiol.* 26 2270–2274. 3148633 10.1128/jcm.26.11.2270-2274.1988 PMC266874

[B46] Sun Y. Dong W. Sun L. Ma L. Shang D. J. (2015). Insights into the membrane interaction mechanism and antibacterial properties of chensinin-1b. *Biomaterials* 37 299–311. 10.1016/j.biomaterials.2014.10.041 25453959

[B47] Wang C. Li H. Li S. Tian L. Shang D. (2012). Antitumor effects and cell selectivity of temporin-1CEa, an antimicrobial peptide from the skin secretions of the Chinese brown frog (*Rana chensinensis*). *Biochimie* 94 434–441. 10.1016/j.biochi.2011.08.011 21871946

[B48] Wu Y. L. Scott E. M. Po A. L. Tariq V. N. (1999). Ability of azlocillin and tobramycin in combination to delay or prevent resistance development in *Pseudomonas aeruginosa*. *J. Antimicrob. Chemother.* 44 389–392. 10.1093/jac/44.3.389 10511408

[B49] Yoon J. Urban C. Terzian C. Mariano N. Rahal J. J. (2004). In vitro double and triple synergistic activities of polymyxin B, imipenem, and rifampin against multidrug-resistant *Acinetobacter baumannii*. *Antimicrob. Agents Chemother.* 48 753–757. 10.1128/aac.48.3.753-757.2004 14982760 PMC353107

[B50] Zasloff M. (2002). Antimicrobial peptides of multicellular organisms. *Nature* 415 389–395. 10.1038/415389a 11807545

[B51] Zhou C. Qi X. Li P. Chen W. Mouad L. Chang M. W. (2010). High potency and broad-spectrum antimicrobial peptides synthesized via ring-opening polymerization of a-amino acid-N-carboxyanhydrides. *Biomacromolecules* 11 60–67. 10.1021/bm900896h 19957992

[B52] Zhou Y. Peng Y. (2013). Synergistic effect of clinically used antibiotics and peptide antibiotics against Gram-positive and Gram-negative bacteria. *Exp. Ther. Med.* 6 1000–1004. 10.3892/etm.2013.1231 24137305 PMC3797290

