# Supplementary figures and images for: Synergistic Antibacterial Activity of Designed Trp-Containing Antibacterial Peptides in Combination With Antibiotics Against Multidrug-Resistant Staphylococcus epidermidis
Source: Front Microbiol. 2019 Nov 25;10:2719. doi: 10.3389/fmicb.2019.02719 (PMC6886405; doi:10.3389/fmicb.2019.02719)

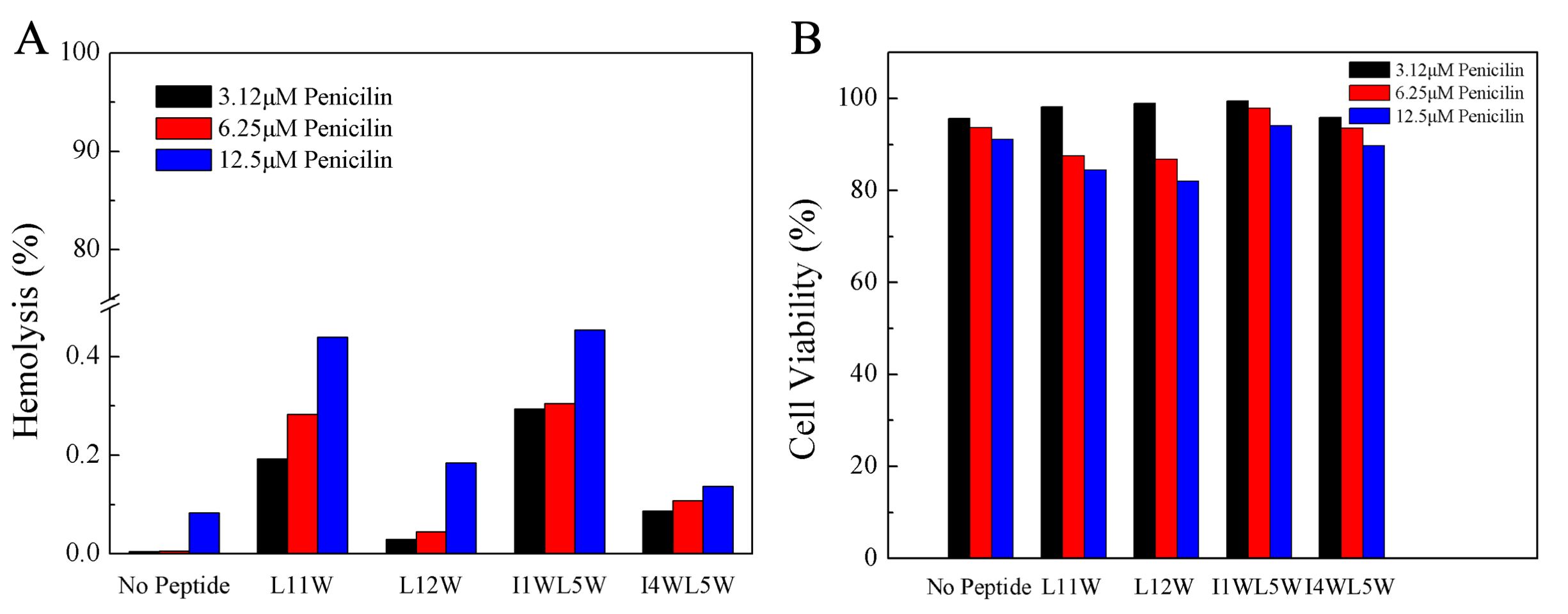

Supplement: FIGURE S1 — Cytotoxicity of the peptides in combination with penicillin. (A) hemolysis on human erythrocyte; (B) Cell viability of human renal epithelial cell 293T. [file Image_1.TIFF]

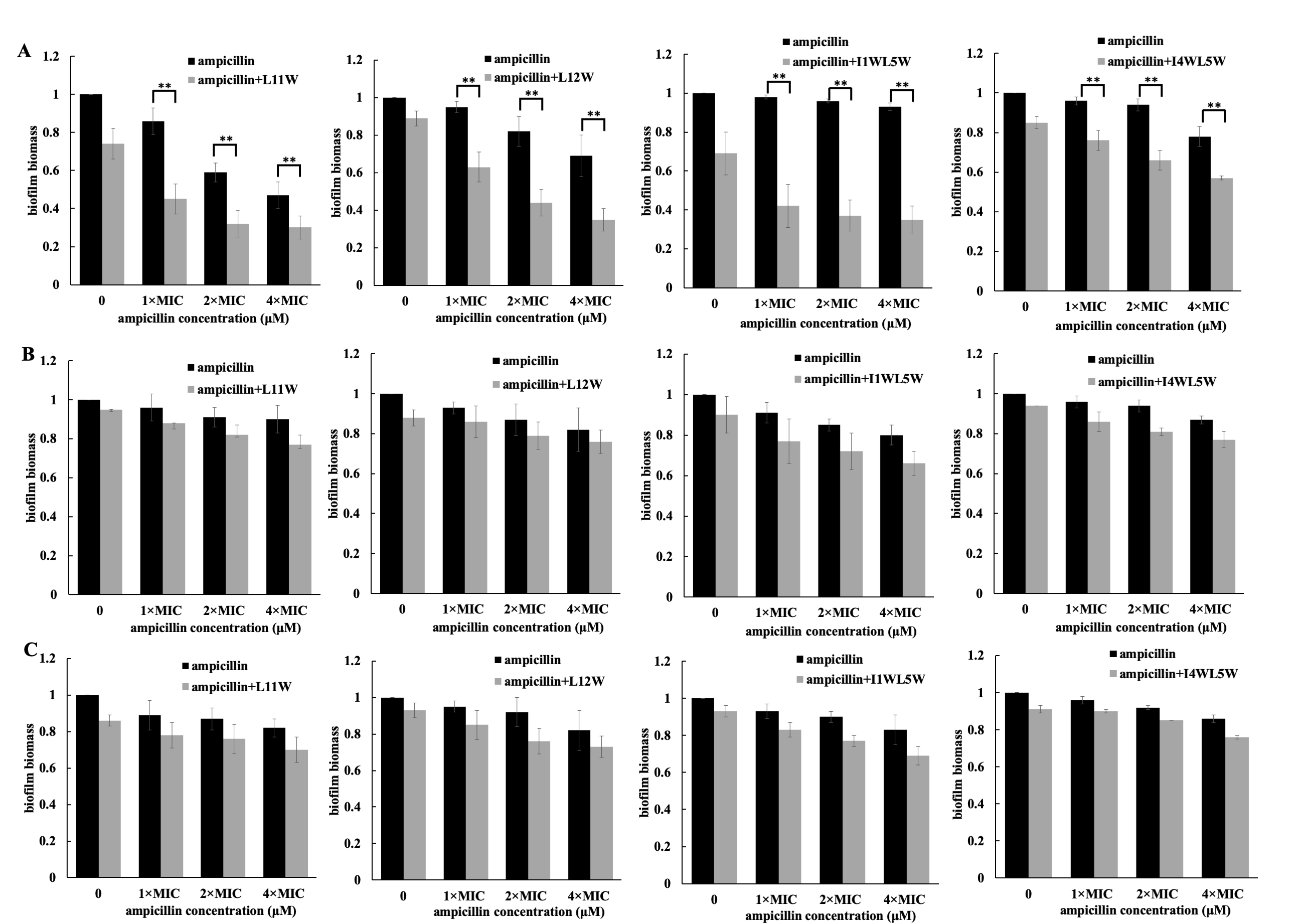

Supplement: FIGURE S2 — Effect of the combination of ampicillin and peptides on the adhesion (A), formation (B) and degradation (C) of biofilm. Log-phase bacteria were incubated with different concentrations of ampicillin in combination with 1/4 × MIC of peptides at 37°C for 1 or 24 h, different concentration of ampicillin as a single drug control and a concentration of ampicillin 0 μM as a nontreated control. After removing the planktonic cells by centrifugation, the biofilms were washed, fixed and stained with crystal violet dye (CV). Biofilm biomass was quantified by using the following formula: OD590 of the sample/OD590 of the nontreated control (the black bar in a concentration of ampicillin 0 μM). The biofilm biomass at 1 and 24 h represents the adhesion and formation of biofilm, respectively. The 24-h mature biofilm of MRSE 1208 cells was prepared as described in the Methods section, and then was treated with different concentrations of ampicillin in combination with 1/4 × MIC of peptides at 37°C for 24 h, different concentration of ampicillin as a single drug control and a concentration of ampicillin 0 μM as a nontreated control. The biofilms were then fixed, stained and quantified as described above. Results represent the average and SEM of at least four independent experiments SEM (∗P < 0.05 and ∗∗P < 0.01). [file Image_2.TIFF]

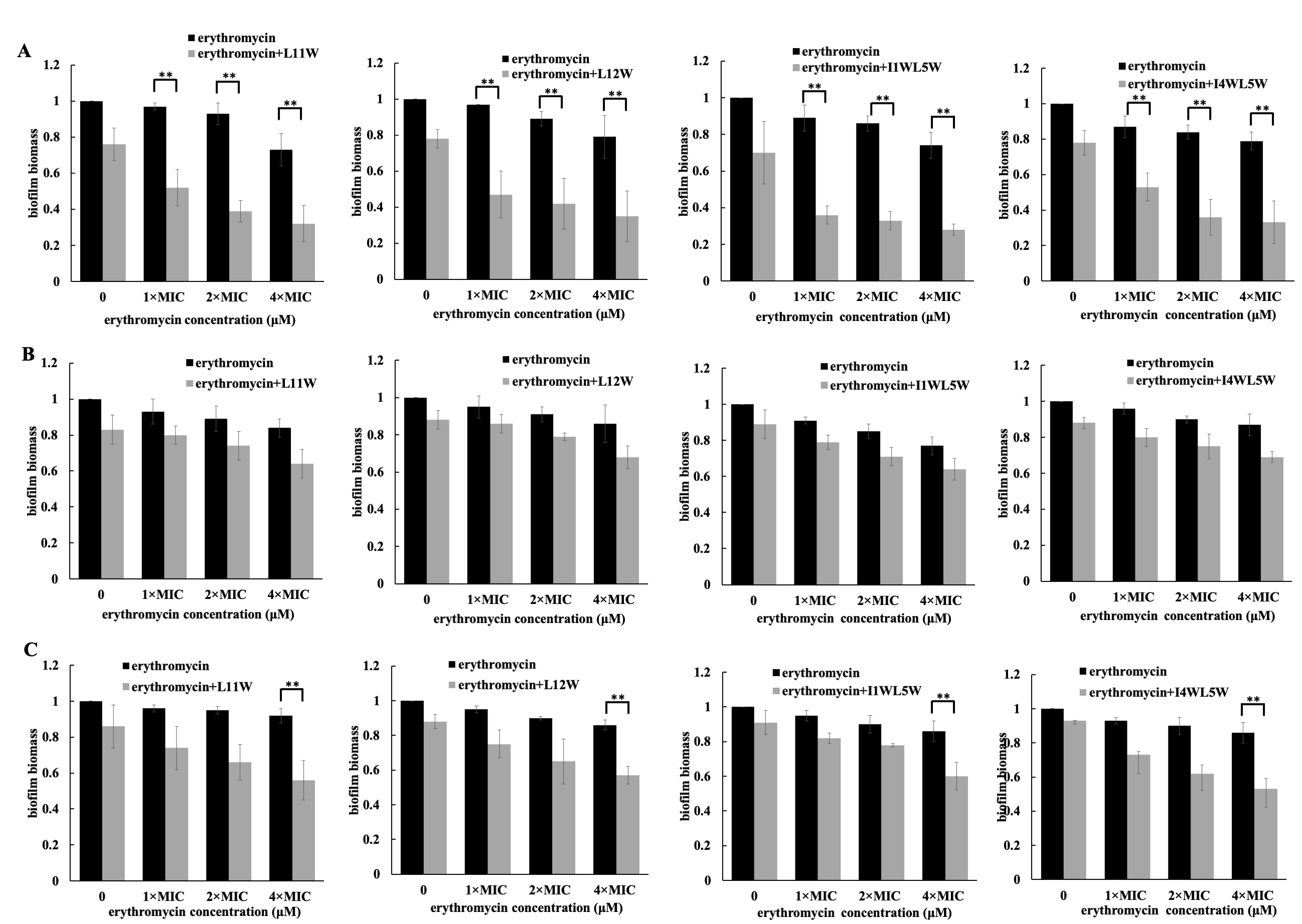

Supplement: FIGURE S3 — Effect of the combination of erythromycin and peptides on the adhesion (A), formation (B) and degradation (C) of biofilm. Log-phase bacteria were incubated with different concentrations of erythromycin in combination with 1/4 × MIC of peptides at 37°C for 1 or 24 h, different concentration of am erythromycin as a single drug control and a concentration of erythromycin 0 μM as a nontreated control. After removing the planktonic cells by centrifugation, the biofilms were washed, fixed and stained with crystal violet dye (CV). Biofilm biomass was quantified by using the following formula: OD590 of the sample/OD590 of the nontreated control (the black bar in a concentration of erythromycin 0 μM). The biofilm biomass at 1 and 24 h represents the adhesion and formation of biofilm, respectively. The 24-h mature biofilm of MRSE 1208 cells was prepared as described in the Methods section, and then was treated with different concentrations of erythromycin in combination with 1/4 × MIC of peptides at 37°C for 24 h, different concentration of erythromycin as a single drug control and a concentration of erythromycin 0 μM as a nontreated control. The biofilms were then fixed, stained and quantified as described above. Results represent the average and SEM of at least four independent experiments SEM (∗P < 0.05 and ∗∗P < 0.01). [file Image_3.TIFF]

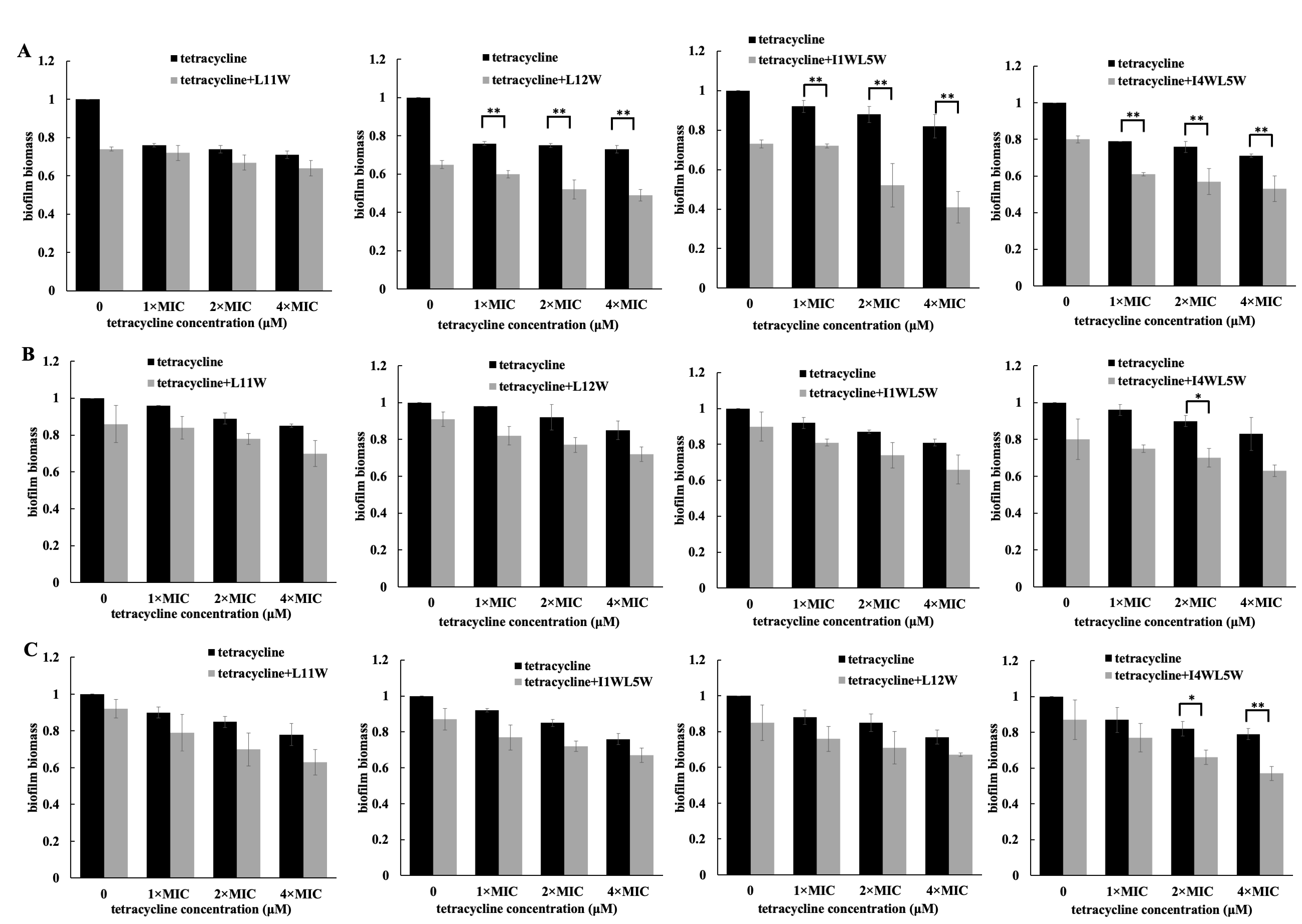

Supplement: FIGURE S4 — Effect of the combination of tetracycline and peptides on the adhesion (A), formation (B) and degradation (C) of biofilm. Log-phase bacteria were incubated with different concentrations of tetracycline in combination with 1/4 × MIC of peptides at 37°C for 1 or 24 h, different concentration of tetracycline as a single drug control and a concentration of tetracycline 0 μM as a nontreated control. After removing the planktonic cells by centrifugation, the biofilms were washed, fixed and stained with crystal violet dye (CV). Biofilm biomass was quantified by using the following formula: OD590 of the sample/OD590 of the nontreated control (the black bar in a concentration of tetracycline 0 μM). The biofilm biomass at 1 and 24 h represents the adhesion and formation of biofilm, respectively. The 24-h mature biofilm of MRSE 1208 cells was prepared as described in the Methods section, and then was treated with different concentrations of tetracycline in combination with 1/4 × MIC of peptides at 37°C for 24 h, different concentration of tetracycline as a single drug control and a concentration of tetracycline 0 μM as a nontreated control. The biofilms were then fixed, stained and quantified as described above. Results represent the average and SEM of at least four independent experiments SEM (∗P < 0.05 and ∗∗P < 0.01). [file Image_4.TIFF]

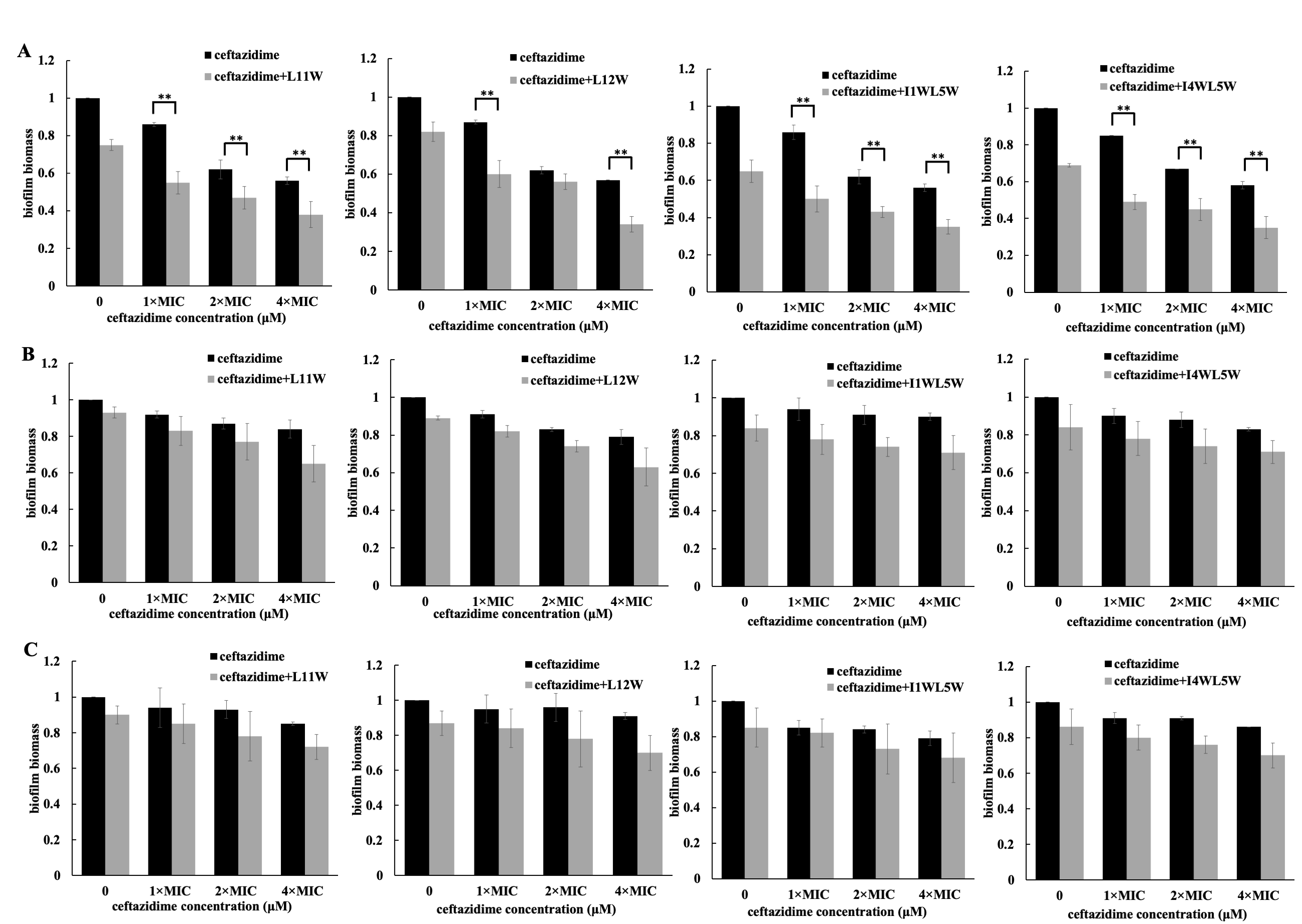

Supplement: FIGURE S5 — Effect of the combination of ceftazidime and peptides on the adhesion (A), formation (B) and degradation (C) of biofilm. Log-phase bacteria were incubated with different concentrations of ceftazidime in combination with 1/4 × MIC of peptides at 37°C for 1 or 24 h, different concentration of ceftazidime as a single drug control and a concentration of ceftazidime 0 μM as a nontreated control. After removing the planktonic cells by centrifugation, the biofilms were washed, fixed and stained with crystal violet dye (CV). Biofilm biomass was quantified by using the following formula: OD590 of the sample/OD590 of the nontreated control (the black bar in a concentration of ceftazidime 0 μM). The biofilm biomass at 1 and 24 h represents the adhesion and formation of biofilm, respectively. The 24-h mature biofilm of MRSE 1208 cells was prepared as described in the Methods section, and then was treated with different concentrations of ceftazidime in combination with 1/4 × MIC of peptides at 37°C for 24 h, different concentration of ceftazidime as a single drug control and a concentration of ceftazidime 0 μM as a nontreated control. The biofilms were then fixed, stained and quantified as described above. Results represent the average and SEM of at least four independent experiments SEM (∗P < 0.05 and ∗∗P < 0.01. [file Image_5.TIFF]
