# Supplementary material for: Synergistic Antibacterial Activity of Designed Trp-Containing Antibacterial Peptides in Combination With Antibiotics Against Multidrug-Resistant Staphylococcus epidermidis
Source: Front Microbiol. 2019 Nov 25;10:2719. doi: 10.3389/fmicb.2019.02719 (PMC6886405; doi:10.3389/fmicb.2019.02719)
Supplement: TABLE S1 — The sequence of primer in this study. [file Table_1.docx]

Table. S1 The sequence of primer in this study

| Name | Sequence（5‘ to 3'） | Length | Genomic Sequence |
| --- | --- | --- | --- |
| *16srRNA*-F  *16srRNA*-R | GCT CGT GTC GTG AGA TGT T  ACT TTA TGG GAT TTG CTT GA | 19  20 | KP728246.1 |
| *Msr*A-F  *Msr*A-R | AAA GGC ACG GTT GAG AAG G  CCT ACA CCA TTT GCA CCT ACG A | 19  22 | EF092840.1 |
| *Tet* m-F  *Tet* m-R | TGG GAT ACG GTA ATA GAG GGA AAC  GAT AAA CAG GGA ACA GGG AAC AA | 24  23 | NC_022618.1 |
| *Mec*A-F  *Mec*A-R | TAT TAG GTT ATG TTG GTC CC  TGT ATG TGC GAT TGT ATT GC | 20  20 | AHA36637.1 |
| *Bla*Z-F  *Bla*Z-R | TCC TAA GGG CCA ATC TGA ACC  ACA CTC TTG GCG GTT TCA CT | 21  20 | NC_013383.1 |

Table S2 The fractional inhibitory concentration of the peptides in the combination and the antibiotics (FIC_a_)

|  | MICs alone(μM) | MICs in the combination and the antibiotics (μM) | | | | | | | | | | FIC_a_ | | | | | | | | | |  |
| --- | --- | --- | --- | --- | --- | --- | --- | --- | --- | --- | --- | --- | --- | --- | --- | --- | --- | --- | --- | --- | --- | --- |
|  |  | Pen | | Amp | | Cef | | Ery | | Tet | | Pen | | Amp | | Cef | | Ery | | Tet | |  |
| L11W | 12.5 | | 3.12 | | 3.12 | | 1.56 | | 3.12 | | 6.25 | | 0.2496 | | 0.2488 | | 0.1248 | | 0.2496 | | 0.5 | |
| L12W | 12.5 | | 3.17 | | 3.12 | | 1.56 | | 3.12 | | 6.25 | | 0.2536 | | 0.2496 | | 0.1248 | | 0.2496 | | 0.5 | |
| I1WL5W | 3.12 | | 0.78 | | 0.78 | | 0.2 | | 0.78 | | 1.56 | | 0.25 | | 0.25 | | 0.0641 | | 0.25 | | 0.25 | |
| I4WL5W | 3.12 | | 0.49 | | 0.39 | | 0.2 | | 0.78 | | 1.56 | | 0.1563 | | 0.125 | | 0.0641 | | 0.25 | | 0.5 | |

FIC_a_ is the MICs of the peptides in the combination /the MICs of the peptides alone.

Pen: Penicillin; Amp: Ampicillin; Cef: Ceftazidime; Ery: Erythromycin; Tet: Tetracycline.

Table S3 The fractional inhibitory concentration of the antibiotics in the combination and the peptides (FIC_b_)

|  | MICs alone(μM) | MICs in the combination and the peptides (μM) | | | | FIC_b_ | | | |
| --- | --- | --- | --- | --- | --- | --- | --- | --- | --- |
|  |  | L11W | L12W | I1WL5W | I4WL5W | L11W | L12W | I1WL5W | I4WL5W |
| Penicillin | 100 | 6.25 | 3.12 | 3.12 | 3.12 | 0.0625 | 0.0312 | 0.0312 | 0.0312 |
| Ampicillin | 100 | 3.12 | 0.78 | 0.78 | 3.12 | 0.0312 | 0.0078 | 0.0078 | 0.0312 |
| Ceftazidime | 50 | 25 | 25 | 25 | 25 | 0.5 | 0.5 | 0.5 | 0.5 |
| Erythromycin | 25 | 0.78 | 0.78 | 0.78 | 1.56 | 0.0312 | 0.0312 | 0.0312 | 0.0624 |
| Tetracycline | 6.25 | 0.78 | 0.39 | 0.2 | 0.78 | 0.1248 | 0.0624 | 0.032 | 0.1248 |

FIC_b_ is the MICs of the antibiotics in the combination /the MICs of the antibiotics alone.
